# Serum Selenium Levels and Lipid Profile: A Systematic Review and Meta-analysis of Observational Studies

**DOI:** 10.1007/s12011-024-04365-4

**Published:** 2024-09-11

**Authors:** Sadegh Mazaheri-Tehrani, Amir Parsa Abhari, Negar Ostadsharif, Arman Shekarian, Mahshad Vali, Elahe Saffari, Kasra Talebi Anaraki, Mohammad Ali Haghighatpanah, Mohammad Fakhrolmobasheri, Marek Kieliszek

**Affiliations:** 1 Child Growth and Development Research Center, Research Institute for Primordial Prevention of Non-Communicable Disease, Isfahan University of Medical Sciences, Isfahan, Iran; 2 Cardiovascular Research Institute, Isfahan University of Medical Sciences, Isfahan, Iran; 3 Student Research Committee, Isfahan University of Medical Sciences, Isfahan, Iran; 4 Heart Failure Research Center, Isfahan Cardiovascular Research Institute, Isfahan University of Medical Sciences, Isfahan, Iran; 5 Isfahan Endocrine and Metabolism Research Center, Isfahan University of Medical Sciences, Isfahan, Iran; 6 School of Medicine, Isfahan University of Medical Sciences, Isfahan, Iran; 7 Department of Cardiovascular Surgery, Chamran Heart Center, Isfahan University of Medical Sciences, Isfahan, Iran; 8 Department of Food Biotechnology and Microbiology, Institute of Food Sciences, Warsaw University of Life Sciences—SGGW, Nowoursynowska 159C, 02-776 Warsaw, Poland

**Keywords:** Lipid profile, Triglycerides, Selenium, Low-density lipoprotein, High-density lipoprotein, Total cholesterol

## Abstract

Selenium is a trace element with pivotal roles in metabolic processes. Studies suggested that selenium deficiency could lead to impaired lipid profiles. However, inconsistent results have been reported regarding the association between serum selenium concentrations and lipid profile (triglycerides, LDL, HDL, VLDL, and total cholesterol). Thus, we aimed to review the correlation between them. A systematic literature search was conducted in PubMed, Embase, Web of Science, Scopus, and Google Scholar until 31 December 2023. The relevant correlation coefficients were used as desired effect sizes to assess the correlation between selenium level and lipid profile. Among 8291 records found in the primary search, 47 and 34 articles were included in the systematic review and meta-analysis, respectively. All included studies were observational investigations and had acceptable quality. Our results failed to reach strong evidence supporting the correlation between serum selenium level and lipid profiles, except for HDL, which showed a weak correlation among both adults (*r* = 0.1 [0.03:0.17]; *I*^2^ = 71%) and pediatrics (*r* = 0.08 [0.03:0.14]; *I*^2^ = 38%). Subgroup analyses based on gender did not reveal a significant or strong correlation with selenium levels (except for total cholesterol in males (*r* = 0.12 [0.01:0.22]; *I*^2^ = 52%)). The results did not change after the sensitivity analysis. Although some previous studies have suggested that selenium deficiency could lead to impaired lipid profile, the findings of this study indicate no strong correlation between serum selenium levels and lipid profile.

**Supplementary Information:**

The online version contains supplementary material available at 10.1007/s12011-024-04365-4.

## Introduction

Selenium is a trace element that is attracting considerable attention due to its potential use in the prevention and treatment of various disease states. This element is crucial in protecting the body against oxidative stress by neutralizing free radicals and reactive oxygen species (ROS). One of the manifestations of the harmful effects of reactive oxygen species is a significant increase in the risk of developing many diseases, including the cardiovascular system [1–3]. Trace elements are necessary for the proper functioning of the body’s physiological mechanisms [4]. Low levels of reactive oxygen species help maintain homeostasis and intracellular signaling processes. Studies have revealed a strong correlation between an imbalance in the quantities of different trace elements and metabolic abnormalities [5–7].

Selenium is used to defend against oxidative damage through antioxidant reactions. Key selenoproteins, such as glutathione peroxidases (GPxs) and thioredoxin reductases, contribute significantly to these protective reactions [8, 9]. Selenium is particularly vital for the function of GPx, an antioxidant enzyme capable of catalyzing the reduction of hydrogen peroxide and lipid hydroperoxides through glutathione metabolism [10]. According to Ruggeri et al. [11], sodium selenite and selenomethionine are able to induce an increase in GPx activity. It has been postulated that selenium may exert anti-atherogenic effects by mitigating oxidative stress in the endothelium [3]. Additionally, selenium used in appropriate doses may have a protective effect in the case of various cancer diseases [12]. The sensitivity of individual cancer cells to the toxic effects of selenium compounds is also different [13]. Therefore, additional studies should be carried out to assess the impact of this element. It is worth noting here that inorganic selenium compounds show almost the same toxicity, while the toxicity of selenium varies significantly in the case of organic selenium compounds [14]. Scientific reports [9, 15, 16] indicate that selenium may support the immune response and the treatment of neurodegenerative diseases. Moreover, an inverse correlation between serum selenium levels and high-sensitive C-reactive protein suggests that low selenium levels may contribute to elevated oxidative stress and lipid peroxidation [17]. According to Gwon et al. [18], selenium protects nerve cells against the adverse effects of lipid peroxidation products (HNE: trans-4-hydroxynonenal). Moreover, selenoproteins and vitamin E inhibit lipid peroxidation processes by reducing hydroperoxides and scavenging lipid peroxide radicals [19]. However, it is worth remembering that an excess of this element is toxic. It is worth noting here that excessive consumption of selenium may have a poisonous effect on the body, and in some cases, it may even be fatal. The recommended daily selenium intake is 1 µg per kg of body weight. Men are advised to consume 70 µg per day, while women should aim for 60 µg per day. Pregnant women should aim for 60 µg per day, and lactating women should aim for 75 µg per day. The optimal serum selenium level is between 130 and 150 ng/mL [8].

Up to date, several meta-analyses have been performed to pool the results of trials regarding the efficacy of selenium supplementation in modulating the lipid profile [20–22]. The latest meta-analysis conducted by Kelishadi et al. [20] on published clinical trials up to December 2021 demonstrates that selenium supplementation did not significantly affect serum levels of triglyceride (TG), low-density lipoprotein cholesterol (LDL-C), and high-density lipoprotein cholesterol (HDL-C), but significantly reduced total cholesterol (TC) levels [20]. On the other hand, several observational studies have evaluated the correlation between serum selenium level and lipid profile, and inconsistent results were reported. Some investigations found no significant association between the serum selenium levels and different lipids [23–25], while others reported a significant association [26–28]. These inconsistent results and findings regarding the impact of selenium supplementation on different lipid levels urged us to conduct a systematic review and meta-analysis of the existing literature to enlighten the proper association of serum selenium with serum lipoproteins. This might aid in adopting preventive and therapeutic strategies to reduce the current burden of cardiovascular and endocrinological disorders. Moreover, it might better clarify the efficacy of selenium supplements in the management of metabolic disorders.

## Methods

### Protocol Registration

The current study was conducted by Preferred Reporting Items for Systematic Reviews and Meta-Analysis (PRISMA) 2020 guidelines [29]. The study protocol has been registered at the International Prospective Register of Systematic Reviews (PROSPERO) with the code CRD42023420741.

### Search Strategy

In order to conduct a comprehensive online search on published paper up to 31 December 2023, we implemented a systematic search in Medline (PubMed), Scopus, Web of Science (WOS), and Embase databases using keywords such as selenium, lipid profile, triglycerides, HDL-C, LDL-C, and total cholesterol. There was no restriction for search in PubMed, WOS, and Embase, but in Scopus, we limited the search terms to title, abstract, and keywords. The exact search line for each database is presented in Supplementary file 1. No language or country restriction was adopted. Google Scholar and the reference lists of relevant review papers were also reviewed to find undetected citations.

### Eligibility Criteria and Study Selection

**Fig. 1:**
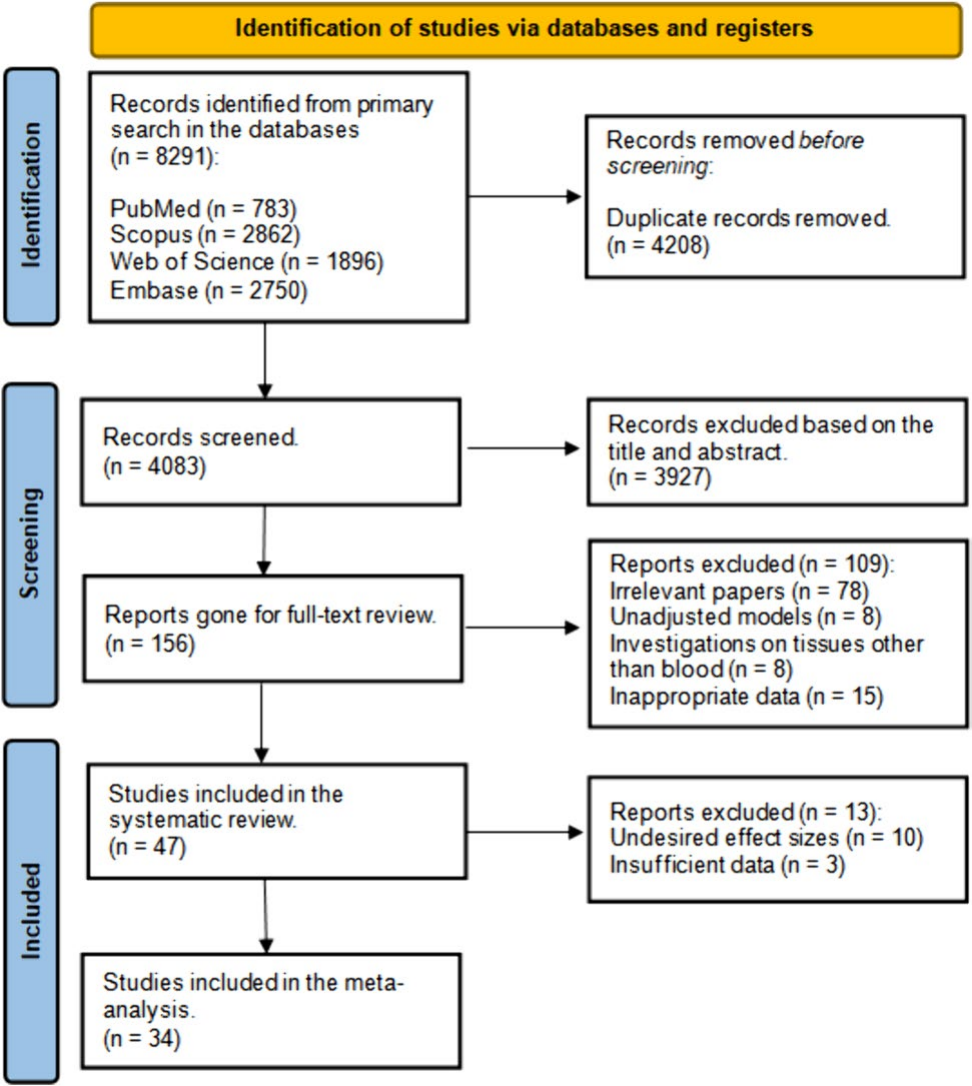
PRISMA flow diagram

Following duplicate records removal, independent reviewers (NO, AS) checked the remaining records for the relevance of the title and abstract to the aims and scope of our study. Meanwhile, conference proceedings, books, letters, and reviews were deleted. Any discrepancies during these steps were resolved by a third reviewer (SMT) or joint consultation. The full text of the chosen studies was retrieved for further screening according to inclusion and exclusion criteria. In case of unavailable full text, the corresponding author of that study was contacted twice with a 10-day interval requesting the full text. Figure 1 shows the eligibility assessment process. We did not use any AI tool to construct the PRISMA flowchart of the study selection or during the screening process.

### Inclusion Criteria

All observational studies that assessed the relationship between serum selenium level (exposure) and lipid profile (TG, TC, HDL-C, LDL-C, and VLDL-C) (outcome) were eligible to be included without the restriction of race, gender, age, and publication date. We included all studies evaluating participants with a healthy state or any underlying disease.

### Exclusion Criteria

Non-English records, animal studies, in vivo and in vitro investigations, and research on selenoprotein levels were excluded. We excluded the studies assessing the correlation between nail selenium levels or urinary levels with lipid parameters.

### Data Extraction

To minimize the potential risk of reporting and data collection bias, data from the included articles were extracted by two independent reviewers (MAH, MV). A pre-designed table with the first author’s name, study design, publication date, location of study, study participants (healthy or any underlying disorder), sample size, gender, age range, BMI, serum selenium level, type of lipid profile, and their serum levels, as well as a summary of main findings as the indices were used to facilitate the data extraction process. The data extraction process was supervised by the third reviewer (MK). In case of incomplete data, the corresponding author of that study was contacted.

### Quality Assessment

To evaluate the methodological quality of the included records, the National Institutes of Health (NIH) quality assessment tool was utilized by two independent reviewers (AS, NO) [30]. There are 14 items on the scale for cross-sectional and cohort studies and 12 items for the case–control studies. Articles are classified as good, fair, or poor. Any controversy was resolved by discussion.

### Statistical Analysis

The study’s findings were synthesized both quantitatively and narratively. To investigate the association between serum selenium level and lipoproteins, including HDL, LDL, TG, and TC, the relevant correlation coefficients accompanied with the included population numbers were extracted from the eligible studies. We have subgrouped the studies according to the age (adults and children) and based on the lipid parameter. Since we ran all the analyses with R version 4.2.3, we did not manually use Fisher r-to-Z transformation for converting our effect sizes into standard average metrics (by default, the metacor function does this transformation automatically) [31, 32]. Forest plots were provided demonstrating pooled coefficients along with the publication year, location, and the participants of the study. We evaluated the between-study heterogeneity using *I*^2^ statistics to discern the suitable type of analysis. In case *I*^2^ statistics results were indicative of substantial heterogeneity (*I*^2^ > 50%), a random effect model was used. We interpreted pooled coefficients as follows: strong correlation (0.5–1, regardless of positivity or negativity), moderate correlation (0.3–0.49), and weak correlation (< 0.29) [33]. Meta-regression was conducted to explore any potential sources of bias. Egger’s test and generated funnel plots were utilized to assess publication bias, both quantitatively and visually. Moreover, we implemented a leave-one-out sensitivity analysis. In this method, we performed meta-analysis by omitting one study at each analysis to determine which studies had affected the result majorly. The statistical significance level was set at *p*-value < 0.05.

## Results

### Study Selection Process

According to the abovementioned search strategy, 8291 records were found in the primary search. After removing duplicates, 4083 items remained. After title and abstract screening, 78 papers were gone for full-text review. No more studies were found in the gray literature or reference checking of relevant review papers. Finally, 47 and 34 articles were included in the systematic review and meta-analysis, respectively.

### Study Characteristics

From 47 included items in the systematic review, 41 articles were conducted in adults’ population, 5 articles in children and adolescents, and 1 article in both groups. Studies investigated the association between serum selenium level and different items of lipid profile including TG, TC, HDL-C, LDL-C, or VLDL-C. Four included studies were conducted in a prospective cohort design, five in a case–control design, and the remaining utilized a cross-sectional manner. These included studies have investigated 40,597 adults and 1226 children or adolescents, from 1985 to 2023. Most of the included studies assessed the relationship in males and females separately or focused on just one gender. Studies that reported correlation coefficients were included in the meta-analysis. The details of included studies are presented in Tables 1 and 2. All included studies have acceptable quality. Supplementary file 2 summarizes the quality assessment process.

**Table 1:** Characteristics of included studies on adults

First author, year	Country	Study design	Population	N subjects (male %)	Age^1^ (year)	BMI^1^	Serum selenium level (µg/L)	Lipid profile	Serum lipid level (mmol/L)	Outcome
Akbaraly 2010^¶^ [34]	France	Prospective cohort	Elderly people with dysglycemia	1162	65	NR	NR	NR	NR	NS
Amirkhizi 2023 [35]	Iran	Cross-sectional	Women with PCOS	125 (0)	33.5 ± 6	28.2 ± 2.9	58.8 ± 9.5	TC	NR	NS
								TG	NR	Significant negative association
								LDL	NR	NS
								HDL	NR	NS
Al-Daghri 2015^§¶^ [36]	Suadi Arabia	Cross-sectional	Healthy adults	24 (100)	28.3 ± 12.9	30.6 ± 6	128.7 ± 33.2	TG	1.3 ± 0.4	NS
								TC	3.5 ± 1	Significant negative association
								HDL	0.86 ± 0.1	NS
				71 (0)	33 ± 10.8	29.7 ± 7.3	91.6 ± 54.4	TG	1.46 ± 0.8	NS
								TC	4.45 ± 1.2	NS
								HDL	1 ± 0.2	Significant negative association
Al-Mubarak 2022^¶^ [26]	The Netherlands	Prospective cohort	Adults	5973 (48)	53.6 ± 12.1	26.7 ± 4.3	84.6 ± 19.5	TC	5.4 ± 1.1	Significant positive association
Arikan 2011^§^ [24]	Turkey	Cross-sectional	Postmenopausal women	107 (0)	54.7 ± 8.8	30.4 ± 3.7	66.7 ± 13.1	TG	1.8 ± 0.6	NS
								TC	5.4 ± 0.9	NS
								HDL	1 ± 0.2	NS
								LDL	3.2 ± 0.7	NS
Barragán 2022^§^ [37]	Multinational	Cross-sectional	Mediterranean population	484 (33)	46.28 ± 13.73	27.87 ± 5.44	94.01 ± 15.01	TC	5.5 ± 1.0	Significant positive association
								TG	1.2 ± 0.7	NS
								HDL	1.5 ± 0.4	Significant positive association
								LDL	3.6 ± 0.8	Significant positive association
Brandt 2021 [38]	USA	Cross-sectional	Adults	7662 (42)	42 ± 21.9	NR	NR	LDL	NR	NS
Bukkens1990 [39]	The Netherlands	Cross-sectional	Adults	82 (84)	59.5 ± 9.5	NR	106.4 ± 23.7	TC	6.5 ± 1.1	NS
								LDL	4.7 ± 1.2	NS
								HDL	1.3 ± 0.3	NS
Cardoso 2024^§^ [25]	Brazil	Cross-sectional	Obese women	84 (0)	33.38 ± 8.59	41.9 ± 6.16	59.93 ± 5.66	TC	5.0 ± 0.8	Significant negative association
								TG	1.6 ± 0.5	NS
								HDL	1.3 ± 0.3	NS
								LDL	3.0 ± 0.8	Significant negative association
								VLDL	0.7 ± 0.2	NS
			Normal weight women	126 (0)	33.56 ± 8.84	22.14 ± 1.66	79.14 ± 7.94	TC	4.98 ± 0.8	NS
								TG	1.4 ± 0.5	NS
								HDL	1.3 ± 0.3	NS
								LDL	3.0 ± 0.7	NS
								VLDL	0.7 ± 0.2	NS
Chen 2023 [40]	China	Cross-sectional	Adults	3548 (57)	51.4 ± 18.76	23.91 ± 15.43	NR	TC	5 ± 19.25	Significant positive association
								TG	1.76 ± 6	Significant negative association
								HDL	2.8 ± 2.2	Significant positive association
								LDL	1.4 ± 1.4	Significant positive association
Christensen 2015 [41]	USA	Cross-sectional	Adults	2287 (48)	37.75 ± 20.7	26.73 ± 6	193 ± 22.89	TC	4.8 ± 1	Significant positive association
								TG	1 ± 0.6	NS
								HDL	1.3 ± 0.3	NS
								LDL	2.9 ± 0.9	Significant positive association
Coudray 1997^§^ [42]	France	Cross-sectional	Pre-aging people	565 (100)	59–71	NR	85 ± 18.27	TC	NR	Significant positive association
								TG	NR	NS
				787 (0)	59–71	NR	95.7 ± 14.82	TC	NR	NS
								TG	NR	NS
El Abd and Aboulsoud 2014^§^ [43]	Egypt	Case–control	T2DM patients and normal cases	90	NR	NR	71.02 ± 8.66	TC	5.5 ± 1.33	Significant negative association
								TG	1.4 ± 0.6	Significant negative association
								HDL	1.2 ± 0.2	NS
								LDL	3.4 ± 0.9	Significant negative association
Ghayour-Mobarhan 2005^§ ¶^ [23]	UK	Cross-sectional	Healthy subjects	189 (50)	49.32 ± 14.59	NR	81.33 ± 15.04	TC	5.4 ± 1.0	NS
								TG	1.1 ± 0.5	NS
								HDL	1.6 ± 0.4	NS
Giacconi 2023^¶^ [44]	International	Cross-sectional	General population	2200 (48)	35–75	110.91 ± 20.69	110.91 ± 20.69	TC	NR	Significant positive association
								TG	NR	Significant positive association
								HDL	NR	Significant positive association
González-Estecha 2017^§^ [28]	Spain	Cross-sectional	Hospital employees	372 (16)	47 ± 10.9	NR	79.5 ± 11.7	TC	5.6 ± 1	Significant positive association
								TG	0.9 ± 0.4	Significant positive association
								LDL	3.4 ± 0.9	Significant positive association
Huang 2020^§^ [45]	USA	Cross-sectional	General population	2903 (49)	61.9 ± 13.7	28.7 ± 5.9	136.4 ± 19.6	TC	5.3 ± 1.1	Significant positive association
								TG	1.8 ± 1.6	Significant positive association
								HDL	1.4 ± 0.4	Significant positive association
								LDL	3.1 ± 1	Significant positive association
Kamal 2009^§¶^ [46]	Egypt	Cross-sectional	T2DM patients and normal cases	65	35–55		65.8 ± 9.4	TC	5.5 ± 1.1	Significant negative association
								TG	1.6 ± 0.9	Significant negative association
								HDL	1.1 ± 0.2	NS
								LDL	3.7 ± 0.9	Significant negative association
Karita 2008^§^ [47]	Japan	Cross-sectional	Premenopausal women	68 (0)	42 ± 4.7	20.8 ± 2.8	134 ± 14	TC	5.2 ± 0.7	NS
								TG	0.7 ± 0.3	NS
								HDL	1.8 ± 0.3	Significant positive association
								LDL	3.3 ± 0.8	NS
			Postmenopausal women	59 (0)	54.9 ± 3.4	21.4 ± 2.9	130 ± 19	TC	5.8 ± 1	NS
								TG	1 ± 0.5	NS
								HDL	1.8 ± 0.3	NS
								LDL	3.7 ± 0.9	NS
Koyama 1995^§¶^ [48]	Japan	Cross-sectional	Middle-aged male office workers (abstainers)	50 (100)	44.6 ± 2.47	22.9 ± 2.09	141.75 ± 17.15	TC	5.6 ± 0.9	NS
								TG	1.2 ± NR	NS
								HDL	1.2 ± 0.2	NS
								LDL	3.9 ± 0.8	NS
			Middle-aged male office workers (alcohol consumers)	49 (100)	43.05 ± 2.59	23.15 ± 2.26	142.7 ± 18.89	TC	5.3 ± 1	NS
								TG	1.4 ± NR	NS
								HDL	1.3 ± 0.4	NS
								LDL	3.2 ± 1	NS
Laird 2015^§¶^ [49]	Canada	Cross-sectional	Healthy adults	2172 (37)	42.1 ± 15.2	28.3 ± 6.5	381 ± 281	HDL	1.5 ± 0.5	Significant positive association
Liu 2011^§¶^ [50]	China	Cross-sectional	Hemodialysis patients	53 (62)	57.2 ± 11.6	NR	93.8 ± 35.16	TC	4.1 ± 0.9	NS
								TG	2.8 ± 1.5	Significant negative association
								HDL	0.8 ± 0.2	Significant positive association
								LDL	3 ± 0.9	Significant negative association
Lu 2019 [51]	Taiwan	Case–control	Adults aged ≥ 40	1165 (35)	65.8 ± 10	25.22 ± 4.2	96.34 ± 25.9	HDL	1.3 ± 0.3	NS
				418 (100)	NR	NR	NR		NR	NS
				747 (0)	NR	NR	NR		NR	NS
Menditto 1995^§^ [52]	Italy	Cross-sectional	Adults	3404 (44)	48.9 ± 13.72	27.96 ± 4.54	91.6 ± 13.39	HDL	3 ± 0.9	Significant positive association
				1520 (100)	48.9 ± 14.05	27.1 ± 3.67	92.2 ± 14.06		1.4 ± 0.3	NS
				1884 (0)	48.9 ± 13.45	28.65 ± 5.04	91 ± 12.81		1.3 ± 0.3	Significant positive association
Molnar 2008^¶^ [53]	Hungary	Cross-sectional	Gestational diabetic and healthy pregnant women at mid-pregnancy and healthy control	61 (0)	30.93 ± 5.73	NR	58.13 ± 20.11	TC	5.7 ± 1.2	Significant negative association
								TG	1.6 ± 0.8	Significant negative association
								LDL	3.4 ± 1	Significant negative association
Mutakin 2013^§^ [54]	Indonesia	Cross-sectional	Obese men	26 (100)	39.7 ± 7.7	28.4 ± 5	95.8 ± 20.2	TG	1.2 ± 0.3	NS
								HDL	1.2 ± 0.2	Significant positive association
			Obese men plus one component of MetS	28 (100)	38.4 ± 5.6	29.3 ± 2.7	102.2 ± 20.7	TG	2.6 ± 1.4	NS
								HDL	1.2 ± 0.2	NS
			Obese men with MetS	24 (100)	36.4 ± 5.8	28.8 ± 2	105.6 ± 22	TG	2.5 ± 1	NS
								HDL	0.9 ± 0.1	NS
Navarro-Alarcon 1998^§^ [55]	Spain	Cross-sectional	Cancer patients	59 (54)	61.6 ± 17.9	NR	54.41 ± 24.8	TC	NR	NS
								TG	NR	NS
Navarro-Alarcon 1999^§¶^ [56]	Spain	Cross-sectional	Patients with acute myocardial infarction	32 (84)	NR	NR	58.67 ± 27.16	TC	NR	NS
								TG	NR	Significant positive association
								HDL	NR	NS
								LDL	NR	NS
			Patients with ischemic cardiomyopathy	50 (76)	NR	NR	55.5 ± 16.7	TC	NR	NS
								TG	NR	NS
								HDL	NR	NS
								LDL	NR	NS
Navarro-Alarcon 2002^§^ [57]	Spain	Cross-sectional	Patients with hepatopathies	50 (62)	44.8 ± 16.8	NR	49.7 ± 15.6	TC	NR	Significant positive association
								TG	NR	NS
Obeid 2008^§^ [27]	Lebanon	Cross-sectional	Adults	398 (40)	18–65	27.18 ± 5.6	141.48 ± 26.1	TC	4.9 ± 1.1	Significant positive association
								TG	1.7 ± 1.4	Significant positive association
								HDL	1.2 ± 0.3	Significant negative association
								LDL	3 ± 1	Significant positive association
Parizadeh 2009^§¶^ [58]	Iran	Cross-sectional	Patients with angiographically defined CAD	152 (65)	54.7 ± 10.1	26.3 ± 5.2	111.8 ± 39.4	TC	NR	NS
								TG	1.6 ± 0.5	NS
								HDL	1.2 ± 0.1	NS
								LDL	3.6 ± 1.2	NS
			Patients with normal angiogram	61 (50)	52.6 ± 11.1	24.9 ± 4.6	112.3 ± 36	TC	NR	NS
								TG	1.2 ± 0.2	NS
								HDL	1.2 ± 0.1	NS
								LDL	3.6 ± 0.7	NS
			Healthy subjects	70 (52)	53.3 ± 10.5	25.2 ± 2.7	104.2 ± 36.4	TC	NR	NS
								TG	1.6 ± 0.7	NS
								HDL	1.2 ± 0.1	NS
								LDL	3.6 ± 0.7	NS
Pemberton 2009^§¶^ [59]	UK	Cross-sectional	Women with rheumatoid arthritis	46 (0)	56 ± 8.14	25.53 ± 4.56	84.55 ± 10.29	TG	1.1 ± 0.5	Significant negative association
								HDL	1.7 ± 0.4	Significant positive association
								LDL	3.1 ± 1.1	NS
			Healthy women	48 (0)	57 ± 6.29	25.16 ± 4.03	91.14 ± 12.72	LDL	3.5 ± 1.2	NS
Peruzzu 2015^§¶^ [60]	Italy	Cross-sectional	T1DM patients	84 (0)	49.7 ± 17.7	NR	133 ± 24	TG	0.8 ± 0.4	Significant positive association
Safarian 2014^§¶^ [61]	Iran	Cross-sectional	Healthy subjects	197 (40)	38.5 ± 16.5	25.43 ± 4.75	116 ± 27.73	TC	4.7 ± 1.3	Significant negative association
								TG	1.4 ± 1	Significant negative association
								HDL	1.0 ± 0.2	NS
								LDL	2.9 ± 0.8	NS
Salonen 1988 [62]	Finland	Cross-sectional	Eastern Finnish men	1132 (100)	54	NR	84.62 ± 20.14	HDL	NR	Significant positive association
Sharma 2023 [63]	India	Case–control	PCOS and healthy women	181 (0)	26.8	23.9	38.3	TC	NR	NS
								HDL	NR	NS
								LDL	NR	NS
Taghavi 2020^§¶^ [64]	Iran	Prospective cohort	Patients with heart failure	55 (60)	50.1 ± 15.4	NR	NR	TC	3.3 ± 0.9	NS
Tinkov 2021^§^ [65]	Poland	Cross-sectional	Obese and normal-weight women	80 (0)	51.95 ± 11.93	29.55 ± 7.79	86.5 ± 23.9	TC	5.1 ± 1.2	Significant positive association
								TG	1.4 ± 0.7	Significant negative association
								HDL	1.6 ± 0.6	Significant positive association
Vidovic 2013^§¶^ [66]	Serbia	Cross-sectional	Patients with schizophrenia	60 (36)	40.1 ± 10.7	27.9 ± 4.9	84.3 ± 27.69	TG	1.7	Significant positive association
								HDL	1.2 ± 0.3	NS
			Healthy subjects	60 (26)	38.7 ± 11.1	24.5 ± 3.3	81.71 ± 15.72	TG	1.2	NS
								HDL	1.5 ± 0.4	NS
Virtamo 1985^§¶^ [67]	Finland	Prospective cohort	Men in a rural area of the eastern part of Finland	527 (100)	55–74	NR	63.3 ± 19.8	TC	NR	Significant positive association
			Men in a rural area of the western part of Finland	582 (100)	55–74	NR	47.5 ± 12.6	TC	NR	Significant positive association
Yang 2010 [68]	Taiwan	Cross-sectional	Elderly population	200 (32)	57.47 ± 9.79	23.42 ± 3.19	90.01 ± 18.16	TC	5.3 ± 1	Significant positive association
								HDL	1.5 ± 0.4	NS
								LDL	3.2 ± 0.7	Significant positive association
Yuan 2015^§^ [69]	China	Case–control	Patients with MetS and healthy subjects	408 (44)	64.05 ± 7.18	24.35 ± 3.72	136.85 ± 64.61	TC	4.9 ± 1.2	Significant positive association
								TG	2 ± 1.5	NS
								HDL	1.4 ± 0.4	Significant positive association
								LDL	2.1 ± 1.4	NS

*Abbreviations: BMI* body mass index, *NS* no significant association, *NR* not reported, *TC* total cholesterol, *TG* triglyceride, *HDL* high-density lipoprotein cholesterol, *LDL* low-density lipoprotein cholesterol, *VLDL* very low-density lipoprotein cholesterol, *T1DM* type 1 diabetes mellitus, *T2DM* type 2 diabetes mellitus, *MetS* metabolic syndrome, *CAD* coronary artery disease, *PCOS* polycystic ovarian syndrome

^1^Values are mean ± standard deviation (SD) if available

^§^Included studies in the meta-analysis

^¶^Studies that evaluated the association between serum selenium level and lipid profile as their secondary outcomes

**Table 2:** Characteristics of included studies on pediatrics

Author, year	Country	Study design	Population	*N* subjects (male %)	Age^1^ (year)	BMI^1^	Serum selenium level (µg/L)	Lipid profile	Serum lipid level (mmol/L)	Outcome
Al-Daghri 2015^§¶^ [36]	Saudi Arabia	Cross-sectional	Healthy children	113 (100)	14.39 ± 1.54	23.38 ± 6.23	114.93 ± 61.04	TG	1.2 ± 0.6	NS
								TC	3.7 ± 0.9	NS
								HDL	0.9 ± 0.2	NS
				146 (0)	14.43 ± 1.57	29.7 ± 7.3	101.09 ± 54.73	TG	1.2 ± 0.6	Significant negative association
								TC	3.6 ± 1.2	NS
								HDL	1 ± 0.2	NS
Azab 2014^§^ [70]	Egypt	Case–control	Obese children	80 (55)	7.8 ± 2.3	28.8 ± 2.6	63.6 ± 15	TG	1.9 ± 0.6	NS
								TC	4.7 ± 1.3	NS
								LDL	2.5 ± 1.2	NS
								HDL	0.8 ± 0.4	NS
			Normal weight children	80 (55)	8.1 ± 1.9	16.2 ± 2.4	78.3 ± 18	TG	0.9 ± 0.3	NS
								TC	2.5 ± 0.7	NS
								LDL	1.9 ± 0.4	NS
								HDL	1.4 ± 0.3	NS
Błażewicz 2015^§¶^ [71]	Poland	Cross-sectional	Obese children	20 (100)	13.1 (6–17)	37.7	82.8 ± 10.3	TC	5.1 ± 0.4	NS
								TG	1.4 ± 0.1	NS
								HDL	1.3 ± 0.2	NS
								LDL	3.1 ± 0.1	NS
				20 (0)	13.1 (6–17)	29.7	80.4 ± 8.2	TC	4.4 ± 0.2	NS
								TG	1.2 ± 0.1	NS
								HDL	1.2 ± 0.3	NS
								LDL	2.6 ± 0.1	NS
			Healthy children	20 (100)	13.5 (8–17)	20.13	111.1 ± 9.5	TC	4.1 ± 0.3	NS
								TG	1 ± 0.2	NS
								HDL	1.4 ± 0.2	NS
								LDL	2.3 ± 0.2	NS
				20 (0)	13.5 (8–17)	20.4	102.3 ± 7.9	TC	3.8 ± 0.3	NS
								TG	0.9 ± 0.1	NS
								HDL	1.5 ± 0.1	NS
								LDL	1.9 ± 0.1	Significant positive association
Gebre-Medhin 1988^§^ [72]	Sweden	Cross-sectional	Healthy children	13	8–17	NT	65 ± 8	TC	4.2 ± 0.8	Significant positive association
								TG	1 ± 0.4	Significant positive association
								HDL	1.3 ± 0.3	NS
Salmonowicz 2014^§¶^ [73]	Poland	Cross-sectional	T1DM children	87 (56)	13 ± 4	NR	58.4 ± NR	TG	1.0 ± NR	NS
								TC	4.5 ± NR	NS
								HDL	1.7 ± NR	NS
								LDL	2.5 ± NR	NS
Spagnolo 1991^§^ [74]	Italy	Cross-sectional	Healthy children	312 (100)	12–13	19.8 ± 3.1	83.89 ± 10.1	TC	4 ± 0.6	Significant positive association
								HDL	1.5 ± 0.3	Significant positive association
				315 (0)	12–13	19.9 ± 3.1	83.89 ± 10.1	TC	4.1 ± 0.6	Significant positive association
								HDL	1.5 ± 0.3	Significant positive association

Abbreviation:* BMI* body mass index, *NS* no significant association, *NR* not reported, *TC* total cholesterol, *TG* triglyceride, *HDL* high-density lipoprotein cholesterol, *LDL* low-density lipoprotein cholesterol, *T1DM* type 1 diabetes mellitus

^1^Values are mean ± standard deviation (SD) if available

^§^Included studies in the meta-analysis

^¶^Studies that evaluated the association between serum selenium level and lipid profile as their secondary outcomes

### *Meta*-analysis of Studies on Adults

**Fig. 2:**
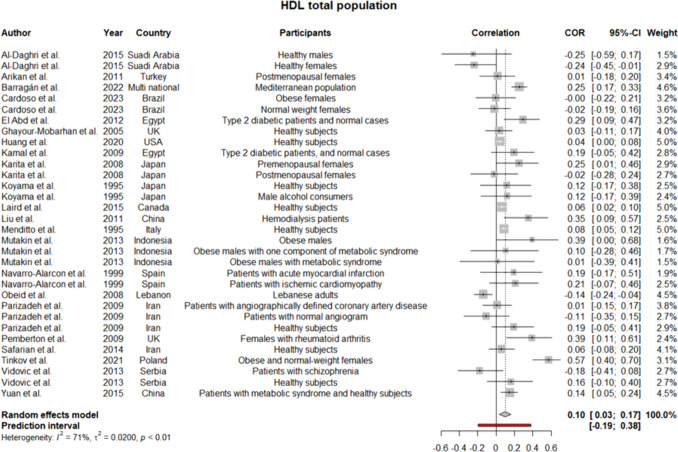
Pooled correlation between HDL-C and serum selenium level in adult population

**Fig. 3:**
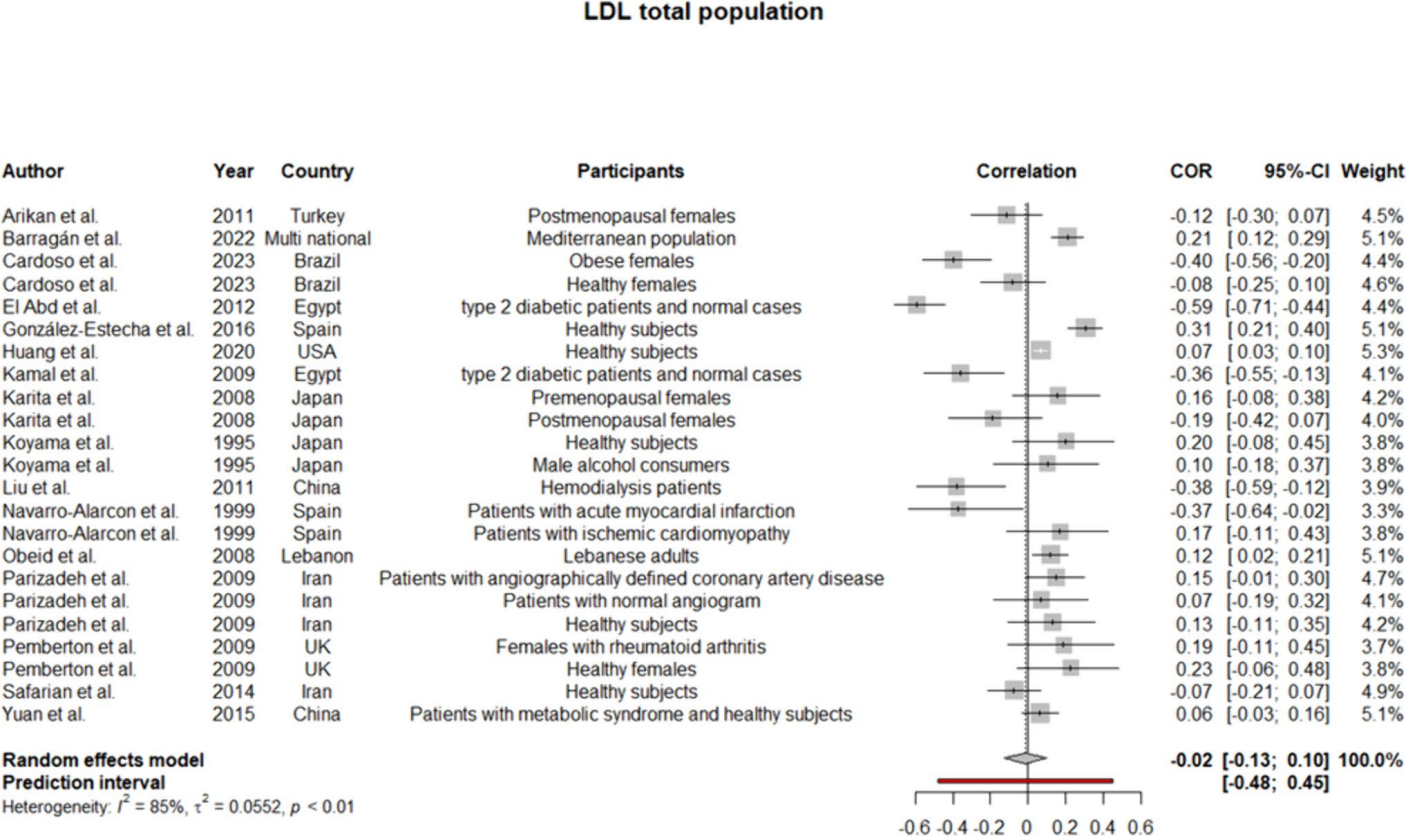
Pooled correlation between LDL-C and serum selenium level in adult population

**Fig. 4:**
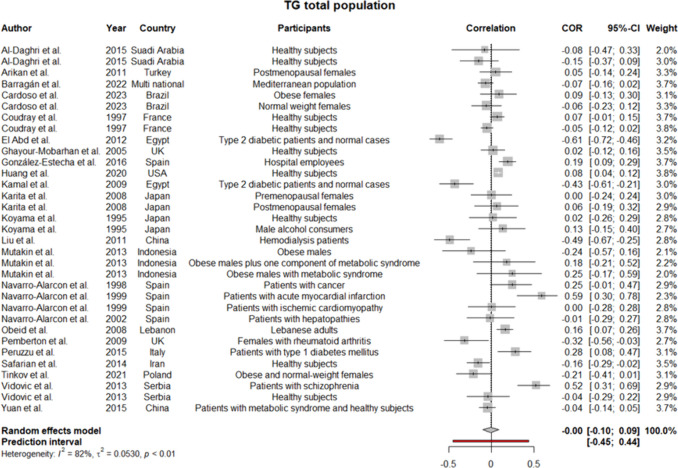
Pooled correlation between TG and serum selenium level in adult population

**Fig. 5:**
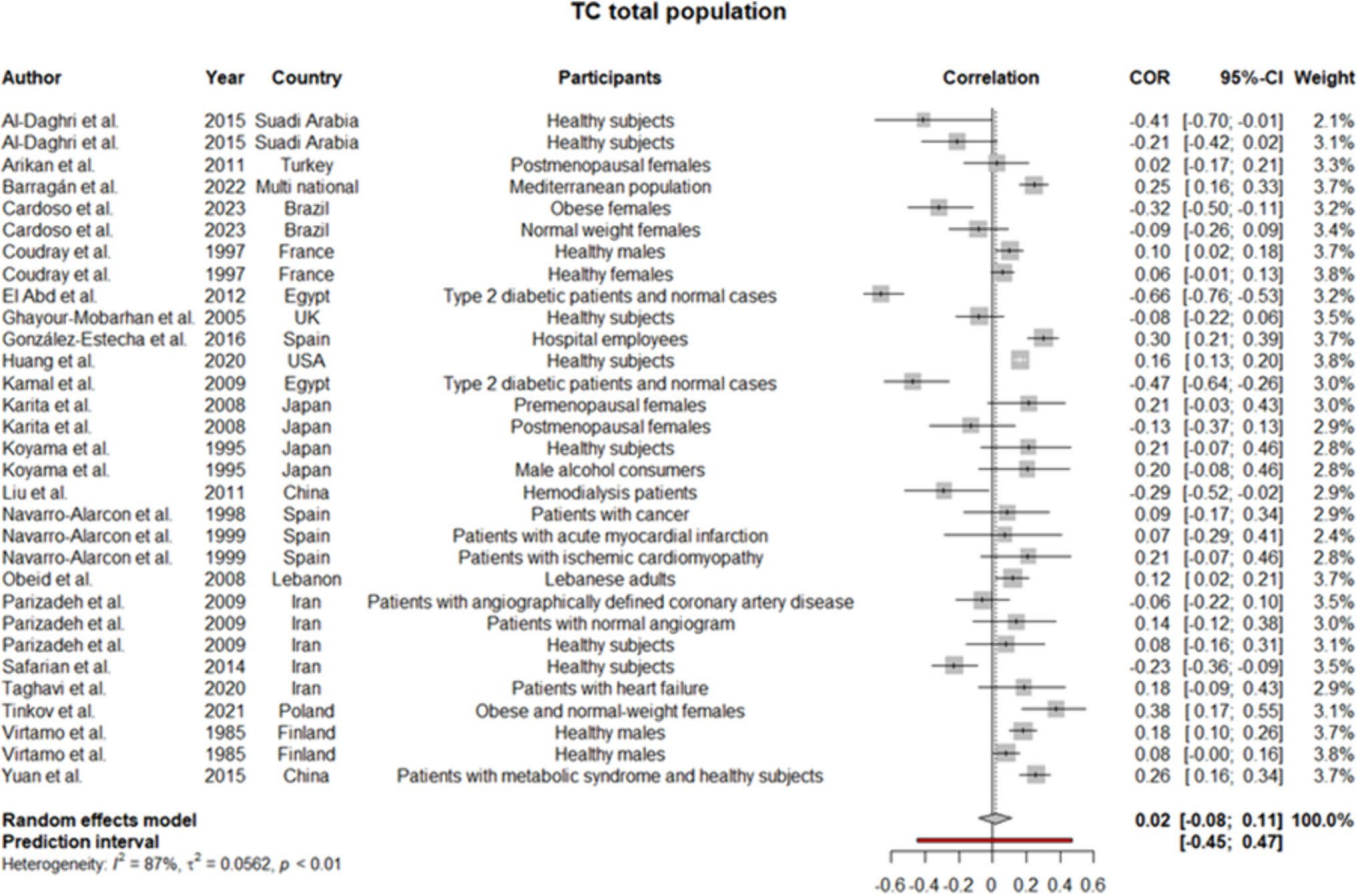
Pooled correlation between TC and serum selenium level in adult population

Regarding the high heterogeneity between the studies, a random effect model was employed for all the analyses. Considering the heterogeneous participants of the research and sex-specific differences of lipoproteins, subgroup analyses were performed for males and females, separately. Pooled coefficients for HDL showed a weak positive correlation between the level of this lipoprotein and serum selenium level (*r* = 0.1 [0.03:0.17]; *p*-value of 0.004) (Fig. 2). The results remained the same but statistically insignificant when analyses were performed for men and women (Supplementary file 3, Fig. S1, S2). Meta-analysis for LDL in the total population showed too weak negative correlation (*r* =  − 0.02 [− 0.13:0.10]) (Fig. 3), which was consistent with the findings in the women subgroup (*r* =  − 0.05 [− 0.26:0.17]) (Supplementary file 3, Fig. S3). Given the low number of studies conducting the analyses on only men, we did not synthesize their data for LDL. Strikingly, our findings regarding TG in total population revealed no correlation with serum selenium content as the pooled *r* was approximately 0 (− 0.10:0.9) (Fig. 4), while in males’ subgroup was 0.07 (− 0.01:0.14) and in females was − 0.02 (− 0.14:0.09) (Supplementary file 3, Fig. S4, S5). Since the *I*^2^ statistics numbers for TG in males and females were not in favor of the random effect model, we repeated the analyses in the fixed effect model. However, the results remained the same (in males were precisely the same, but in females, the new *r* along with a 95% confidence interval was − 0.03 [− 0.08:0.02]). Meta-analysis of 22 studies assessing the correlation of TC with selenium level displayed pooled *r* of 0.02 (− 0.08:0.11) (Fig. 5). Of note, the results for males were indicative of a weak positive correlation, which was statistically significant (*r* = 0.12 [0.01:0.22]; *p*-value of 0.03); however, the results for females were not similar (*r* =  − 0.01 [− 0.20:0.18]) (Supplementary file 3, Fig. S6, S7).

### *Meta*-analysis of Studies on Pediatrics

**Fig. 6:**
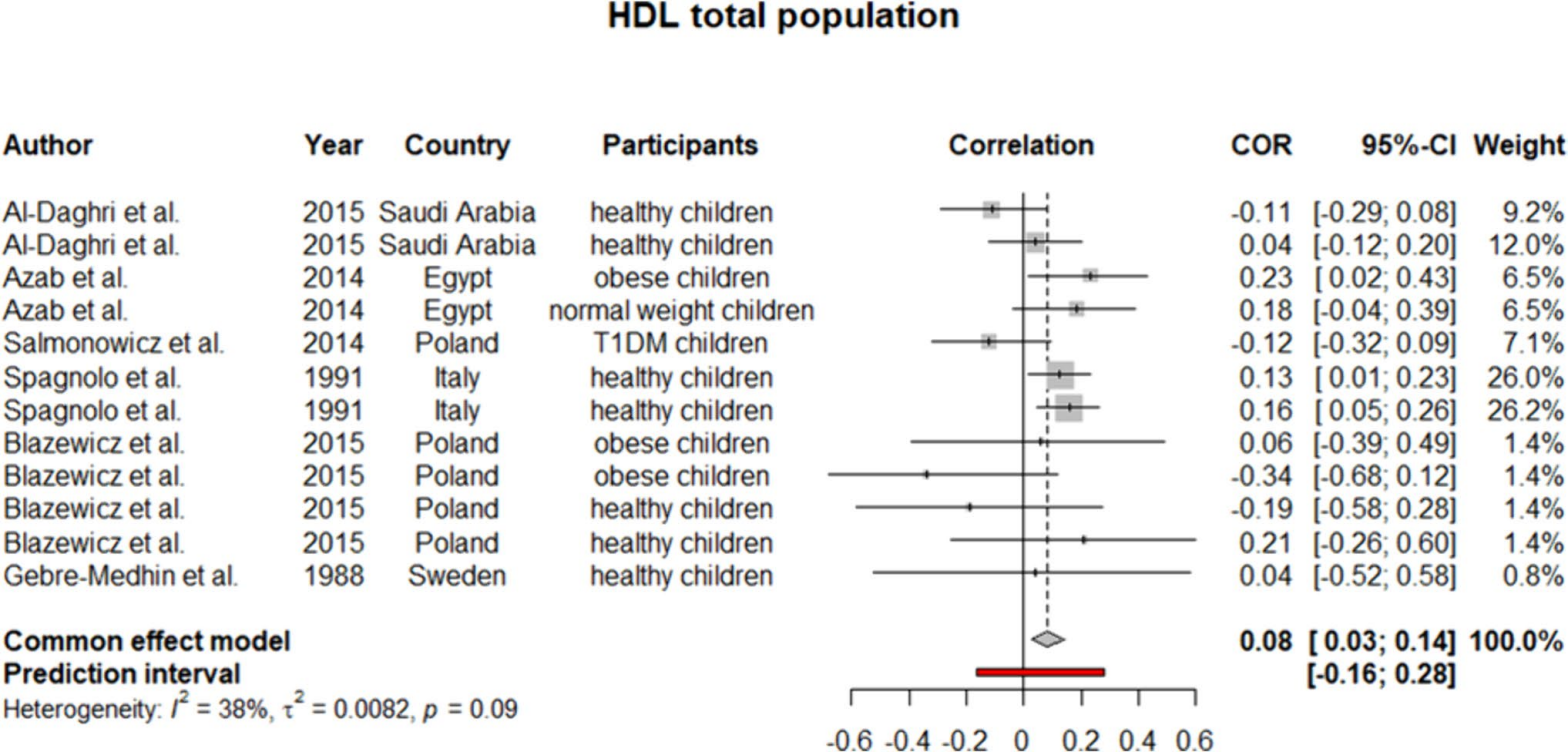
Pooled correlation between HDL-C and serum selenium level in pediatrics population

**Fig. 7:**
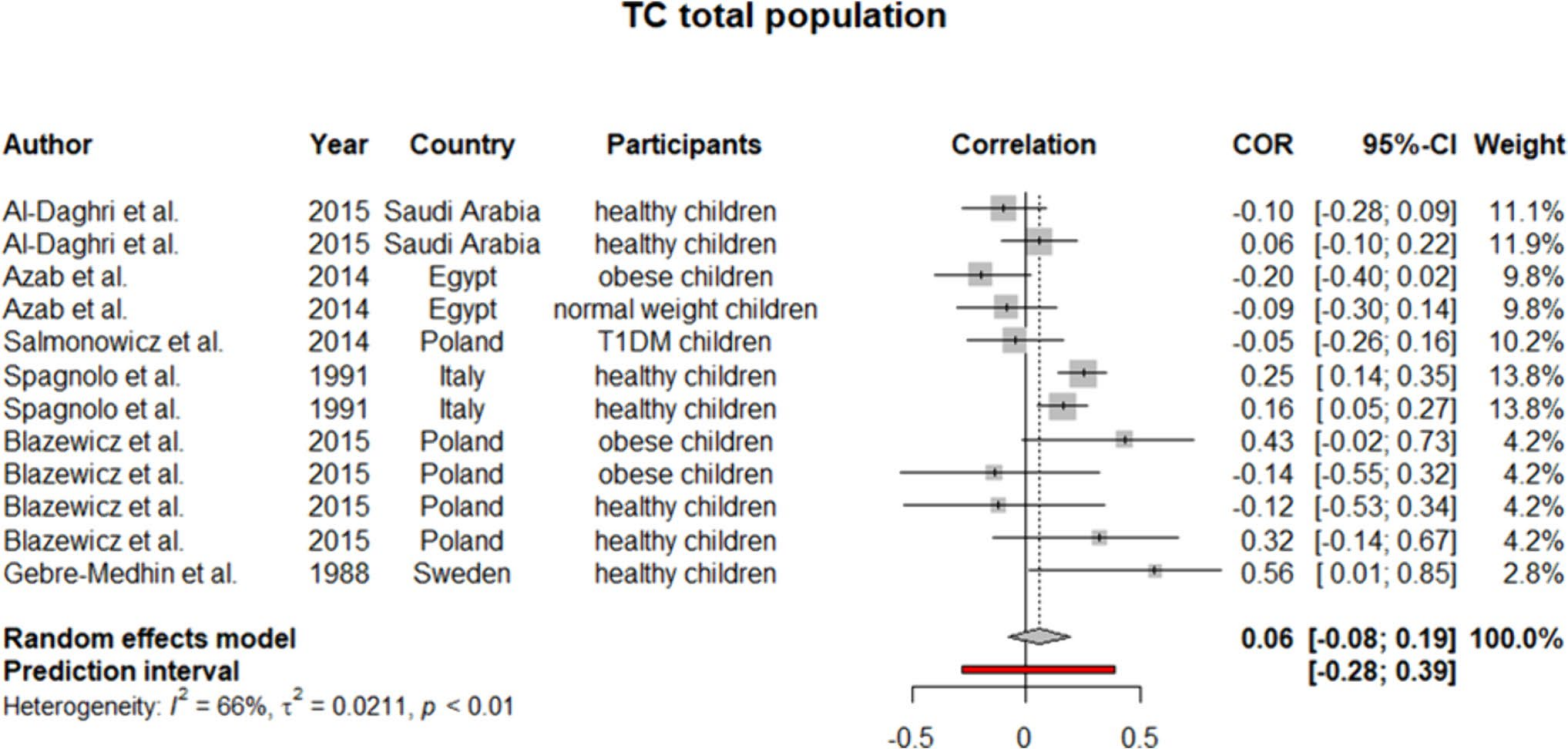
Pooled correlation between TC and serum selenium level in pediatrics population

**Fig. 8:**
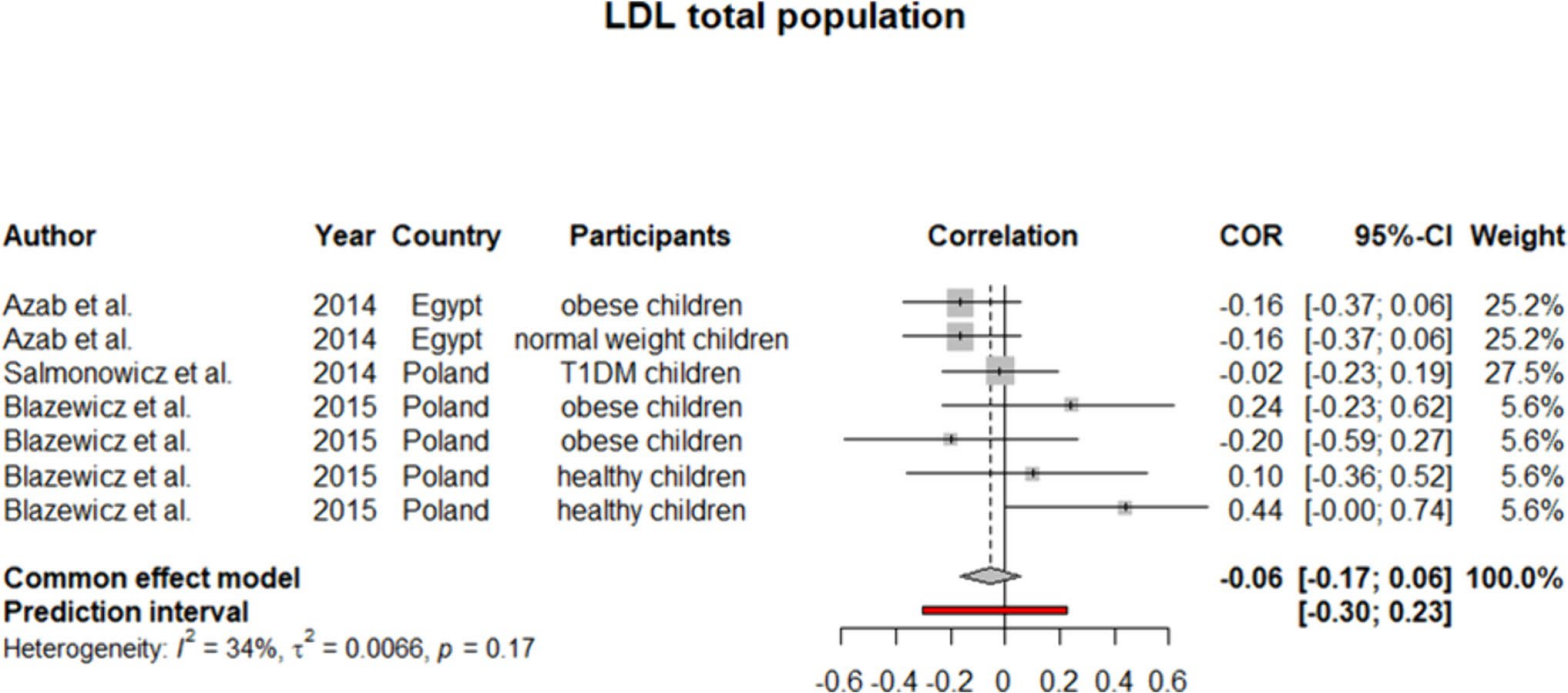
Pooled correlation between LDL-C and serum selenium level in pediatrics population

**Fig. 9:**
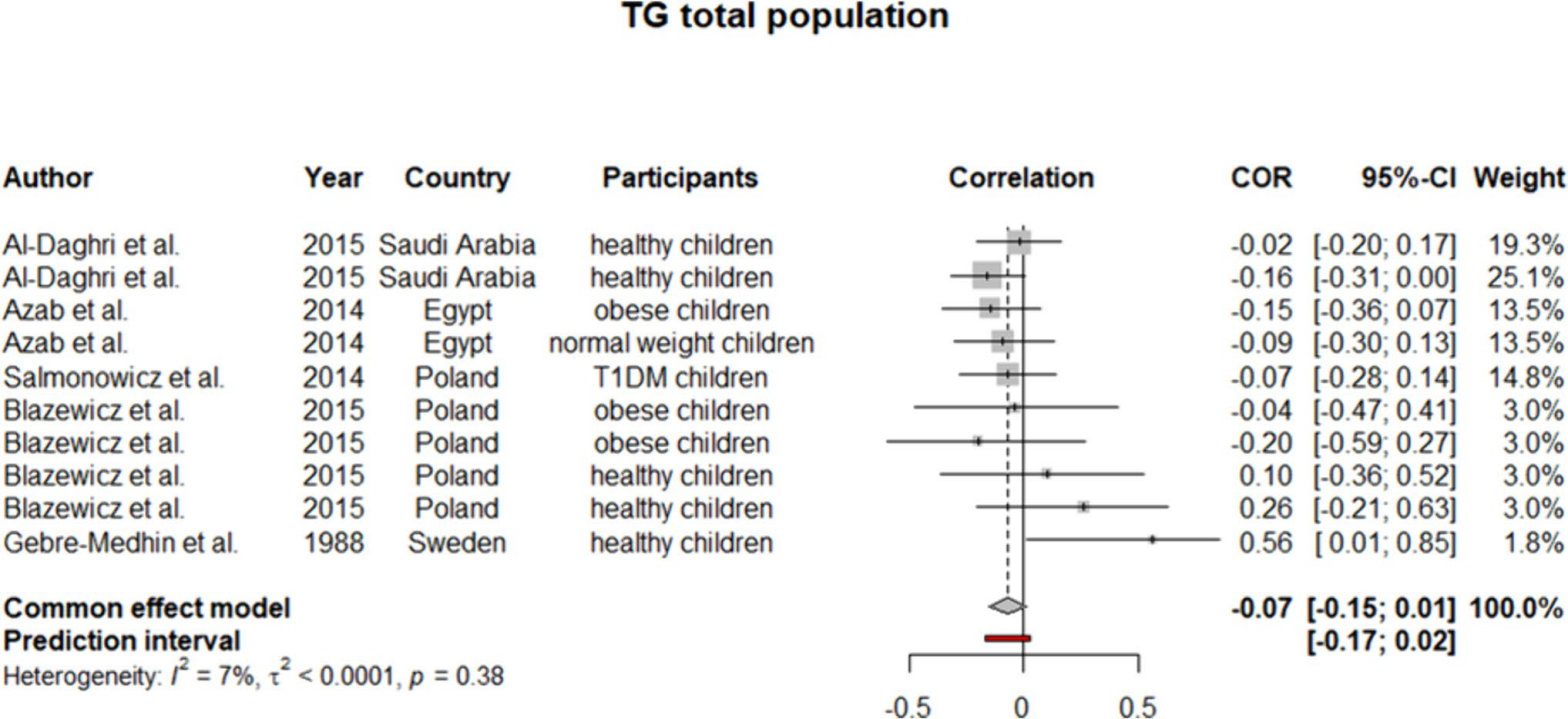
Pooled correlation between TG and serum selenium level in pediatrics population

To ensure the generalizability of this study’s results, we further performed a meta-analysis on subjects younger than 18 years old. Six studies were identified as eligible to be included in the synthesis. Considering *I*^2^ statistics results for heterogeneity in these six studies, a fixed effect model was used (except for TC). For HDL, our analysis yielded a pooled *r* of 0.08 (0.03:0.14) (Fig. 6). Similarly, pooled *r* for TC showed a weak positive but insignificant correlation with selenium level (*r* = 0.06 [− 0.08:0.19]) (Fig. 7). On the other side, as demonstrated in Figs. 8 and 9, an insignificant, weak negative correlation was detected for LDL and TG, respectively.

### Sensitivity Analysis

To examine whether the findings were overshadowed by a single study, we performed a leave-one-out sensitivity analysis. As displayed in Supplementary file 3 (Figs. S8–S11), this analysis confirmed the stability of our results.

### *Meta*-regression

We carried out meta-regression to explore the potential association between mean age, mean BMI of the participants, and the country in which the study was conducted and our outcome. We did not perform meta-regression on males and females separately, as we only did the analyses on the total population. Countries’ distribution for HDL and TC, mean age for TC, and mean BMI for LDL could indeed influence our effect size significantly.

### Publication *Bias*

As noted earlier, Egger’s test was utilized to assess publication bias. Similar to meta-regression, Egger’s test was only implemented on the total population, regardless of sex. Publication bias was only significant for TC (intercept of − 2.05 and standard error of 0.7; *p*-value of 0.01) (Supplementary file 3, Fig. S12); as in TG, HDL, and LDL, publication bias was evident neither in Egger’s test nor in funnel plots (Supplementary file 3, Figs. S13–S15).

## Discussion

Selenium is a vital microelement found in proteins like GPX, thioredoxin reductase, and other selenoproteins. It has numerous pivotal roles, such as antioxidant and anti-inflammatory properties, and its deficiency was found to be associated with various complications such as cardiovascular diseases, neoplasms, and Keshan disease. It may also lead to disease susceptibility and poor health maintenance [8, 75]. Serum selenium concentration and its association with metabolism and metabolic factors are an active area of research. In recent years, a growing body of evidence has emerged, showing this remarkable association and even proposing the pharmacological and nutritional implications of selenium for various disorders. Among these metabolic factors, plasma lipoproteins have been a matter of high concern and interest. Hence, we sought to investigate the correlation between serum selenium content and HDL, LDL, TG, and TC as, to the best of our knowledge, this is the first aggregate data meta-analysis aimed to do so. Although there is one meta-analysis on the relationship between trace elements and dyslipidemia [76], they did not assess each lipid parameter separately. Besides, they utilized mean differences and standard deviations as the desired effect size and evaluated serum trace element levels between the two groups. According to the analyses, we failed to reach strong evidence supporting this correlation; except for HDL in both pediatrics and adults, the pooled estimates for the other lipoproteins were suggestive of approximately no correlation. Even for HDL, the pooled coefficient showed a weak correlation (less than 0.3). Identical to these findings, subgroup analyses on males and females did not reveal a significant or intense (or even moderate) correlation with selenium level (except for TC in males). We assumed that high heterogeneity among the studies was the reason for these findings. However, sensitivity analyses confirmed the stability of our results. Moreover, even in the analyses with lower *I*^2^ heterogeneity and through a fixed effect model, the findings were not discordant. To date, few meta-analyses have assessed the effect of selenium supplementation on lipid profile, and inconsistent results have been reported [20–22, 77]. The latest meta-analysis conducted on different databases up to December 2021 found a significant reduction in just TC levels [20]. However, selenium and probiotic co-supplementation demonstrated beneficial effects on lipid profile [78]. These controversies in the association between selenium intake or its serum levels with lipid profile might be due to its narrow therapeutic window [20].

Different confounding factors have been found to affect the association between selenium status and lipid profile. Menopausal status and estrogen levels affect the antioxidant balance, and in many cases, after a decline in estrogen levels, an increase in lipid peroxidation and ROS formation was observed [79]. Among diabetes patients, a more significant association between selenium concentrations and lipid profile was observed, which is supposed to be due to insulin resistance [80]. The studies that have investigated diabetic individuals in our review also reported the same results [43, 46]. This association was also found to be affected by smoking status, being hypertensive, and alcohol consumption [80]. In the following, we proceeded to discuss another factor that impacts this relationship.

In line with our results, a systematic review conducted on published literature till May 2014 demonstrated considerable controversy surrounding the relation between serum selenium level and metabolic syndrome (MetS) [17]. However, recent investigations suggested that higher serum selenium levels are an independent risk factor for MetS, primarily through impairments in glycemic profile. Two included studies have assessed the relationship between serum selenium levels and lipid profiles among MetS cases and have reported controversial results [54, 69].

Polycystic ovarian syndrome (PCOS) is a prevalent endocrine disorder among young women found to be associated with metabolic disorders such as impaired lipid profile [81, 82]. Women with PCOS are suggested to have lower levels of plasma selenium, which correlates negatively with LH and total testosterone, which suggests that selenium may contribute to the development of PCOS related to hyperandrogenism. However, selenium supplementation did not significantly improve BMI, weight, cholesterol, and testosterone levels among PCOS patients [83]. Two of the included studies investigated the relationship between serum selenium levels and lipid parameters among PCOS cases [35, 63], and only TG levels were reported to be associated with selenium levels [35].

Different demographical factors such as age and sex might affect the serum selenium levels and even their association with lipid parameters. Studies have reported contradictory results regarding the association between selenium levels and age, which might be due to the different age ranges of their samples and even the selenium contents of their diet [28]. The results of our study demonstrated that age affects the association between serum selenium levels and TC. The existing literature on selenium levels and gender differences, in most cases, suggested higher selenium levels among males [36, 84–86]. Results of our subgroup analysis, based on gender, demonstrated changes in the associations that might be due to higher dietary intake among males [87]. A few studies demonstrated a lower selenium level in postmenopausal women due to an estrogen-deficit state [47, 88]. Another study reported controversial results [28]. According to Colpo et al. [89], consumption of up to 50 g of Brazil nuts may have a positive effect on the lipid profile by reducing the level of LDL-C while increasing the concentration of HDL-C in the blood serum.

Selenium enters the food chain via plants that take it up from the soil. On the other hand, there is an uneven distribution in the selenium content of soil. Thus, the selenium concentration of foods varies geographically, and as a result, it causes variation in the selenium level of people [90, 91]. The results of our meta-regression found that the region of the participants affects the association between selenium status and lipid profile. Therefore, another critical factor that might affect the level of selenium is the country of residency [92]. Unfortunately, none of the included studies mentioned the selenium content in their locations and its association with lipid parameters. Further, epidemiological investigations are needed in this regard. Of note, selenium supplements should be consumed carefully as there is a narrow range between a safe and harmful amount of this element.

Results of our study among pediatric populations demonstrated a significant positive association between serum selenium level and HDL, the same as adults. Studies on children and adolescents also suggested significantly lower selenium levels among obese children [70, 71]. Children up to 6 years are recommended to intake selenium for an amount of 6–22 µg per day (depending on their body weight and age) [93].

### Strengths and Limitations

Our study has some noteworthy strengths. Firstly, we decided not to restrict our study to a particular age group to increase the comprehensiveness of our research. Secondly, we analyzed data separately for men and women to see if there were gender differences in how selenium level affects their lipid profile. Additionally, the leave-one-out sensitivity analysis indicated the robustness of our findings. However, some limitations also exist in our study. Firstly, despite our comprehensive search strategy, Egger’s test suggested the potential existence of publication bias regarding the association of serum selenium and TC. Secondly, due to insufficient data and heterogeneous reporting of odds ratios in the original studies, we could not pool the odds ratios. Thirdly, some studies evaluated the association between serum selenium level and lipid profile as their primary outcome, while others investigated it as their secondary outcome. This issue might affect the results of their measurements.

## Conclusion

Our results indicated a weak correlation just between serum selenium level and HDL-C among both adults and pediatrics. The pooled effect sizes for the other lipoproteins were suggestive of approximately no correlation. Subgroup analyses on males and females did not reveal a significant or remarkable correlation with selenium levels (except for TC in males). Although previous studies have demonstrated that selenium deficiency could lead to impaired lipid profile [22], according to the findings of this study and earlier studies on selenium supplementation [22, 77], it seems that there is no strong correlation between selenium status and lipid profile.

## Supplementary Information

Below is the link to the electronic supplementary material.

Supplementary file1 (DOCX 14 KB)

Supplementary file2 (DOCX 27 KB)

Supplementary file3 (DOCX 322 KB)

## Data Availability

No datasets were generated or analysed during the current study.
