# Supplementary material for: Serum Selenium Levels and Lipid Profile: A Systematic Review and Meta-analysis of Observational Studies
Source: Biol Trace Elem Res. 2024 Sep 11;203(5):2517–38. doi: 10.1007/s12011-024-04365-4 (PMC12125032; doi:10.1007/s12011-024-04365-4)
Supplement: Supplementary file 1 — Supplementary file1 (DOCX 14 KB) [file 12011_2024_4365_MOESM1_ESM.docx]

Supplementary file 1

Search Line for each database

PubMed: (783 records no filter)

(Selenium OR "Selenium"[Mesh] OR Selen*) AND (“serum lipid profile*” OR “lipid profile*” OR “lipid level*” OR “triglyceride*” OR “Triglycerides”[Mesh] OR “total cholesterol” OR “high-density lipoprotein cholesterol” OR “Cholesterol, HDL”[Mesh] OR “HDL” OR “low-density lipoprotein cholesterol” OR “Cholesterol, LDL”[Mesh] OR “LDL” OR “very low-density lipoprotein cholesterol” OR “Cholesterol, VLDL”[Mesh] OR “VLDL” “cholesterol” OR “Cholesterol”[Mesh])

Scopus: (2862 records title abstract keywords)

(Selenium OR Selen*) AND (“serum lipid profile*” OR “lipid profile*” OR “lipid level*” OR “triglyceride*” OR “total cholesterol” OR “high-density lipoprotein cholesterol” OR “HDL” OR “low-density lipoprotein cholesterol” OR “LDL” OR “very low-density lipoprotein cholesterol” OR “VLDL” OR “cholesterol”)

Embase: (2750 records, all fields)

(Selenium OR Selen*) AND (‘serum lipid profile*’ OR ‘lipid profile*’ OR ‘lipid level*’ OR ‘triglyceride*’ OR ‘total cholesterol’ OR ‘high-density lipoprotein cholesterol’ OR ‘HDL’ OR ‘low-density lipoprotein cholesterol’ OR ‘LDL’ OR ‘very low-density lipoprotein cholesterol’ OR ‘VLDL’ OR ‘cholesterol’)

WOS: (1896 records, no filter)

(Selenium OR Selen*) AND (“serum lipid profile*” OR “lipid profile*” OR “lipid level*” OR “triglyceride*” OR “total cholesterol” OR “high-density lipoprotein cholesterol” OR “HDL” OR “low-density lipoprotein cholesterol” OR “LDL” OR “very low-density lipoprotein cholesterol” OR “VLDL” OR “cholesterol”)
