# Supplementary material for: Serum Selenium Levels and Lipid Profile: A Systematic Review and Meta-analysis of Observational Studies
Source: Biol Trace Elem Res. 2024 Sep 11;203(5):2517–38. doi: 10.1007/s12011-024-04365-4 (PMC12125032; doi:10.1007/s12011-024-04365-4)
Supplement: Supplementary file 2 — Supplementary file2 (DOCX 27 KB) [file 12011_2024_4365_MOESM2_ESM.docx]

***Supplementary file 2***

***Details of quality assessment process***

| **Study** | **Items of NIH quality assessment tool^*^** | | | | | | | | | | | | | | **Summary quality** |
| --- | --- | --- | --- | --- | --- | --- | --- | --- | --- | --- | --- | --- | --- | --- | --- |
|  | **1** | **2** | **3** | **4** | **5** | **6** | **7** | **8** | **9** | **10** | **11** | **12** | **13** | **14** |  |
| Akbaraly et al. 2010 | yes | yes | yes | yes | no | yes | yes | yes | yes | yes | yes | NA | no | yes | Good |
| Al-daghri et al. 2015 | yes | yes | yes | no | no | no | no | yes | yes | no | yes | NA | NA | no | Fair |
| Al-mubarak et al. 2021 | yes | yes | yes | yes | no | no | yes | NA | yes | no | yes | NA | yes | yes | Good |
| Amirkhizi et al. 2023 | yes | yes | yes | yes | yes | no | no | NA | yes | no | yes | NA | NA | yes | Good |
| Arikan et al. 2011 | yes | no | yes | yes | no | no | no | NA | yes | no | yes | NA | NA | no | Fair |
| Berragan et al. 2022 | yes | yes | yes | yes | no | no | no | NA | yes | no | yes | NA | NA | yes | Good |
| Blazewicz et al. 2015 | yes | yes | yes | yes | no | no | no | NA | yes | no | yes | NA | NA | yes | Fair |
| Brandt et al. 2023 | yes | yes | yes | yes | no | no | no | NA | yes | yes | yes | NA | NA | yes | Good |
| Buckens et al. 1990 | yes | yes | yes | yes | no | no | no | NA | yes | no | yes | NA | NA | yes | Fair |
| Cardoso et al. 2023 | yes | yes | yes | yes | no | no | no | NA | yes | no | yes | NA | NA | yes | Good |
| Chen et al. 2023 | yes | yes | yes | yes | no | no | no | NA | yes | no | yes | NA | NA | yes | Good |
| Christensen et al. 2015 | yes | yes | yes | yes | no | no | no | NA | yes | no | yes | NA | NA | yes | Good |
| Coudray et al. 1997 | yes | yes | yes | yes | NA | yes | no | NA | yes | yes | yes | NA | NA | no | Good |
| Gebre-Medhin et al. 1988 | yes | yes | yes | no | no | no | no | NA | yes | no | yes | NA | NA | no | Fair |
| Ghayour- mobarhan et al. 2005 | yes | yes | yes | yes | no | no | no | NA | yes | no | yes | NA | NA | yes | Fair |
| Giacconi et al. 2023 | yes | yes | yes | yes | NA | no | no | NA | yes | no | yes | NA | NA | yes | Fair |
| Gonzalez- Estecha et al.2016 | yes | yes | yes | yes | yes | no | no | NA | yes | no | yes | NA | NA | yes | Good |
| Huang et al. 2019 | yes | yes | yes | yes | yes | no | no | NA | yes | no | yes | NA | NA | yes | Good |
| Karita et al. 2008 | yes | yes | yes | yes | yes | no | no | NA | yes | no | yes | NA | NA | yes | Good |
| Kamal et al. 2009 | yes | yes | yes | yes | no | no | no | NA | yes | no | yes | NA | NA | no | Fair |
| Koyama et al. 1995 | yes | yes | yes | yes | no | no | no | NA | yes | no | yes | NA | NA | no | Fair |
| Laird et al. 2015 | yes | yes | yes | yes | no | no | no | NA | yes | no | yes | NA | NA | yes | Good |
| Liu et al. | yes | yes | yes | yes | no | no | no | NA | yes | no | yes | NA | NA | no | Fair |
| Menditto et al. 1995 | yes | yes | yes | yes | yes | no | no | NA | yes | no | yes | NA | NA | yes | Fair |
| Molnar et al. 2007 | yes | yes | yes | yes | no | no | no | NA | yes | no | yes | NA | NA | no | Fair |
| Mutakin et al. 2013 | yes | yes | yes | yes | no | no | no | NA | yes | no | yes | NA | NA | no | Fair |
| Navarroalarcon et al. 1998 | yes | yes | yes | yes | no | no | no | NA | yes | no | yes | NA | NA | yes | Good |
| Navarroalarcon et al. 1999 | yes | yes | yes | yes | no | no | no | NA | yes | no | yes | NA | NA | yes | Good |
| Navarroalarcon et al. 2002 | yes | yes | yes | yes | no | no | no | NA | yes | no | yes | NA | NA | yes | Good |
| Obeid et al. 2008 | yes | yes | yes | yes | no | no | no | NA | yes | no | yes | NA | NA | no | Fair |
| Parizadeh et al. 2008 | yes | yes | yes | yes | no | no | no | NA | yes | no | yes | NA | NA | no | Fair |
| Pemberton et al. 2009 | yes | yes | yes | yes | no | no | no | NA | yes | no | yes | NA | NA | no | Good |
| Peruzzu et al. 2015 | yes | yes | yes | yes | no | no | no | NA | yes | no | yes | NA | NA | no | Fair |
| Safarian et al. 2014 | yes | yes | yes | yes | no | no | no | NA | yes | no | yes | NA | NA | yes | Fair |
| salmonowicz et al. 2011 | yes | yes | yes | yes | no | no | no | NA | yes | no | yes | NA | NA | no | Fair |
| Salonen et al. 1988 | yes | yes | yes | yes | no | no | no | NA | yes | no | yes | NA | NA | yes | Fair |
| spagnolo et al. 1991 | yes | yes | yes | yes | no | no | no | NA | yes | no | yes | NA | NA | yes | Fair |
| Taghavi et al. 2020 | yes | yes | yes | yes | no | no | no | NA | yes | yes | yes | NA | yes | no | Fair |
| Tinkov et al. 2021 | yes | yes | yes | yes | no | no | no | NA | yes | no | yes | NA | NA | yes | Good/ Fair |
| Vidovic et al. 2013 | yes | yes | yes | yes | no | no | no | NA | yes | no | yes | NA | NA | no | Fair |
| Virtamo et al. 1985 | yes | yes | yes | yes | no | no | yes | yes | yes | yes | yes | no | no | yes | Good |
| Yang et al. 2010 | yes | yes | yes | yes | no | no | no | NA | yes | no | yes | NA | NA | yes | Fair |
| Azab et al. 2012 | yes | yes | no | yes | yes | yes | yes | NA | NA | yes | yes | no |  |  | Good |
| El abd et al. 2012 | yes | yes | yes | yes | yes | yes | yes | NA | NA | yes | yes | no |  |  | Good |
| Lu et al. 2019 | yes | yes | yes | yes | yes | yes | yes | NA | no | no | yes | no |  |  | Good |
| Sharma et al 2023 | yes | yes | yes | yes | yes | yes | yes | NA | no | yes | yes | no |  |  | Good |
| Yuan et al. 2015 | yes | yes | yes | yes | yes | yes | yes | yes | no | yes | no | yes |  |  | Good |
| ^*^ NIH quality assessment tool has 12 questions for Case-Control studies and 14 questions for observational Cohort and Cross-sectional studies. (Available online at: <https://www.nhlbi.nih.gov/health-topics/studyquality-assessment-tools>)  NA: not available | | | | | | | | | | | | | | | |
