# Supplementary material for: Serum Selenium Levels and Lipid Profile: A Systematic Review and Meta-analysis of Observational Studies
Source: Biol Trace Elem Res. 2024 Sep 11;203(5):2517–38. doi: 10.1007/s12011-024-04365-4 (PMC12125032; doi:10.1007/s12011-024-04365-4)
Supplement: Supplementary file 3 — Supplementary file3 (DOCX 322 KB) [file 12011_2024_4365_MOESM3_ESM.docx]

**Supplementary file 3**

**Figure S1.** Pooled correlation between HDL-C and serum selenium level in males.

**Figure S2.** Pooled correlation between HDL-C and serum selenium level in females.

**Figure S3.** Pooled correlation between LDL-C and serum selenium level in females.

**Figure S4.** Pooled correlation between TG and serum selenium level in males.

**Figure S5.** Pooled correlation between TG and serum selenium level in females.

**Figure S6.** Pooled correlation between TC and serum selenium level in males.

**Figure S7.** Pooled correlation between TC and serum selenium level in females.

**Figure S8.** Leave-one-out sensitivity analysis correlation between HDL-C and serum selenium level among adults.

**Figure S9.** Leave-one-out sensitivity analysis correlation between LDL-C and serum selenium level among adults.

**Figure S10.** Leave-one-out sensitivity analysis correlation between TG and serum selenium level among adults.

**Figure S11.** Leave-one-out sensitivity analysis correlation between TC and serum selenium level among adults.

**Figure S12.** Funnel plot for publication bias of correlation between TC and serum selenium level among adults.

**Figure S13.** Funnel plot for publication bias of correlation between LDL-C and serum selenium level among adults.

**Figure S14.** Funnel plot for publication bias of correlation between TG and serum selenium level among adults.

**Figure S15.** Funnel plot for publication bias of correlation between HDL-C and serum selenium level among adults.


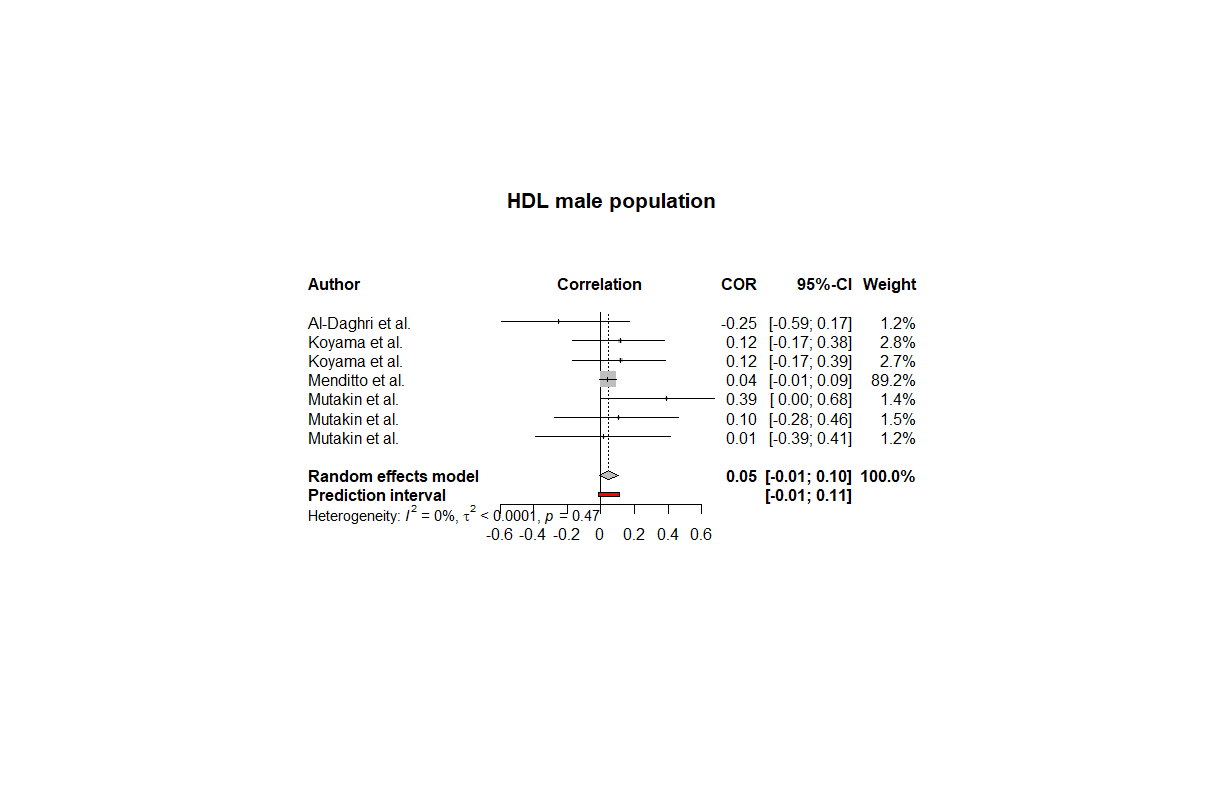


**Figure S1.** Pooled correlation between HDL-C and serum selenium level in males.


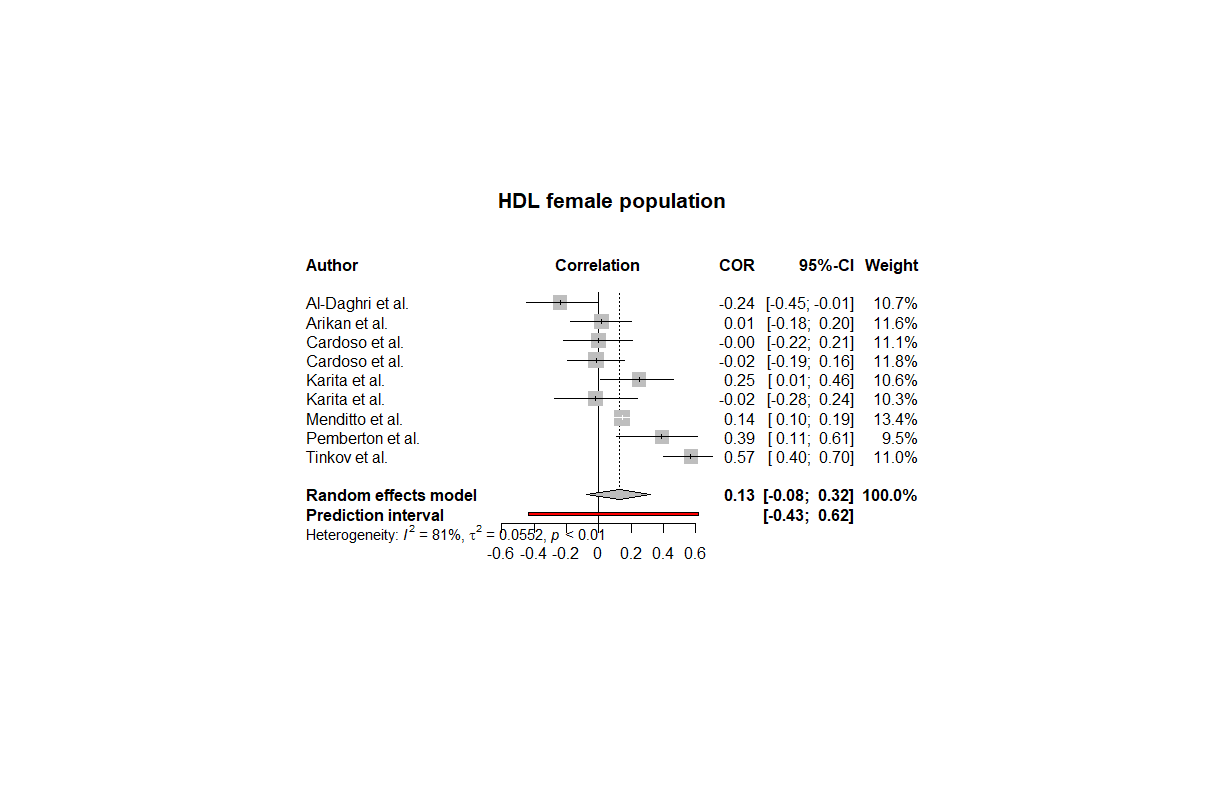


**Figure S2.** Pooled correlation between HDL-C and serum selenium level in females.


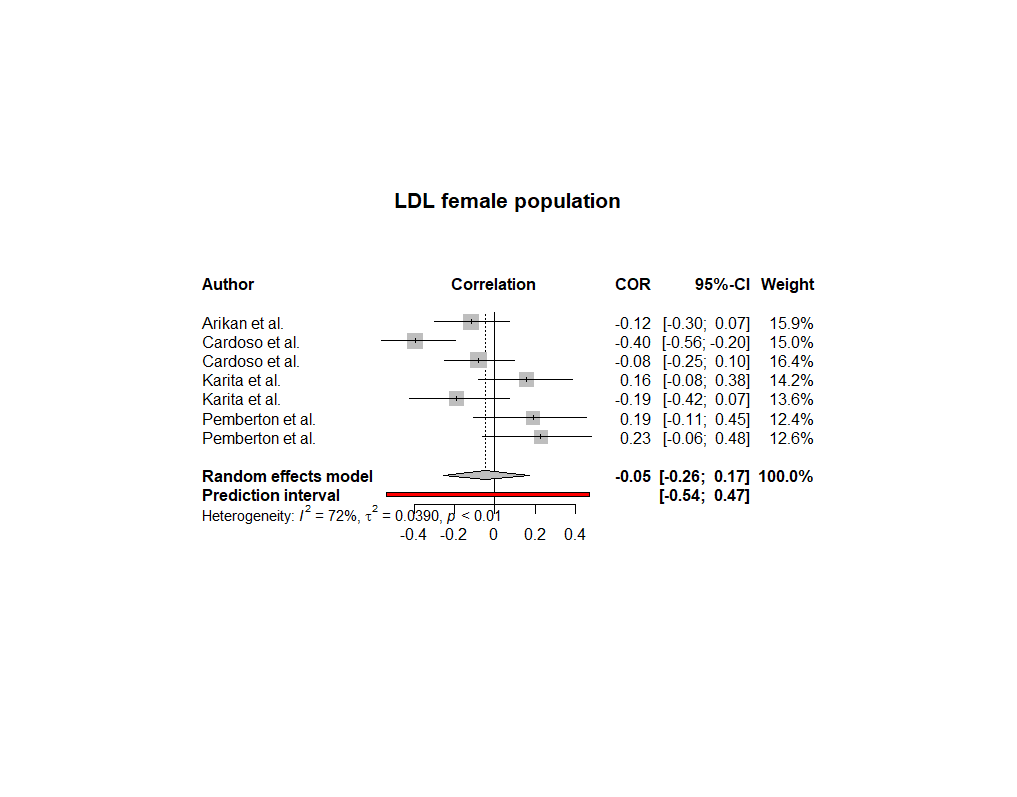


**Figure S3.** Pooled correlation between LDL-C and serum selenium level in females.


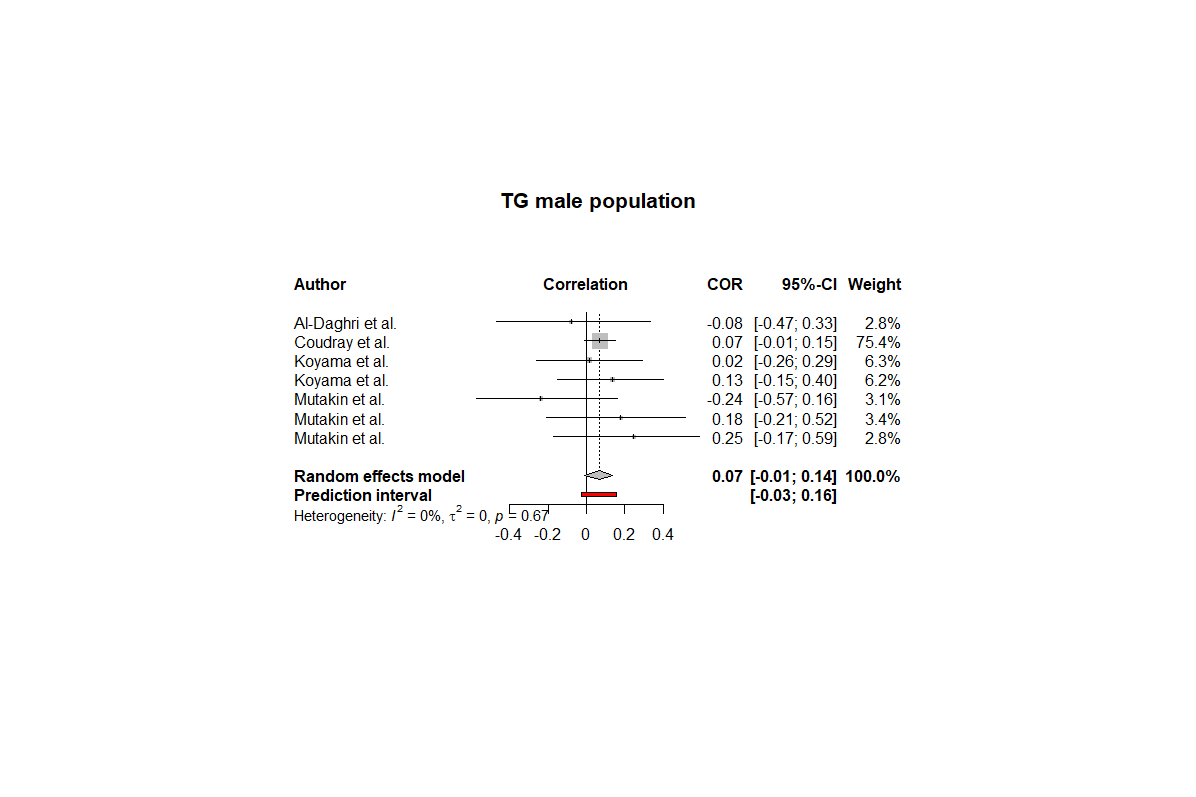


**Figure S4**. Pooled correlation between TG and serum selenium level in males.


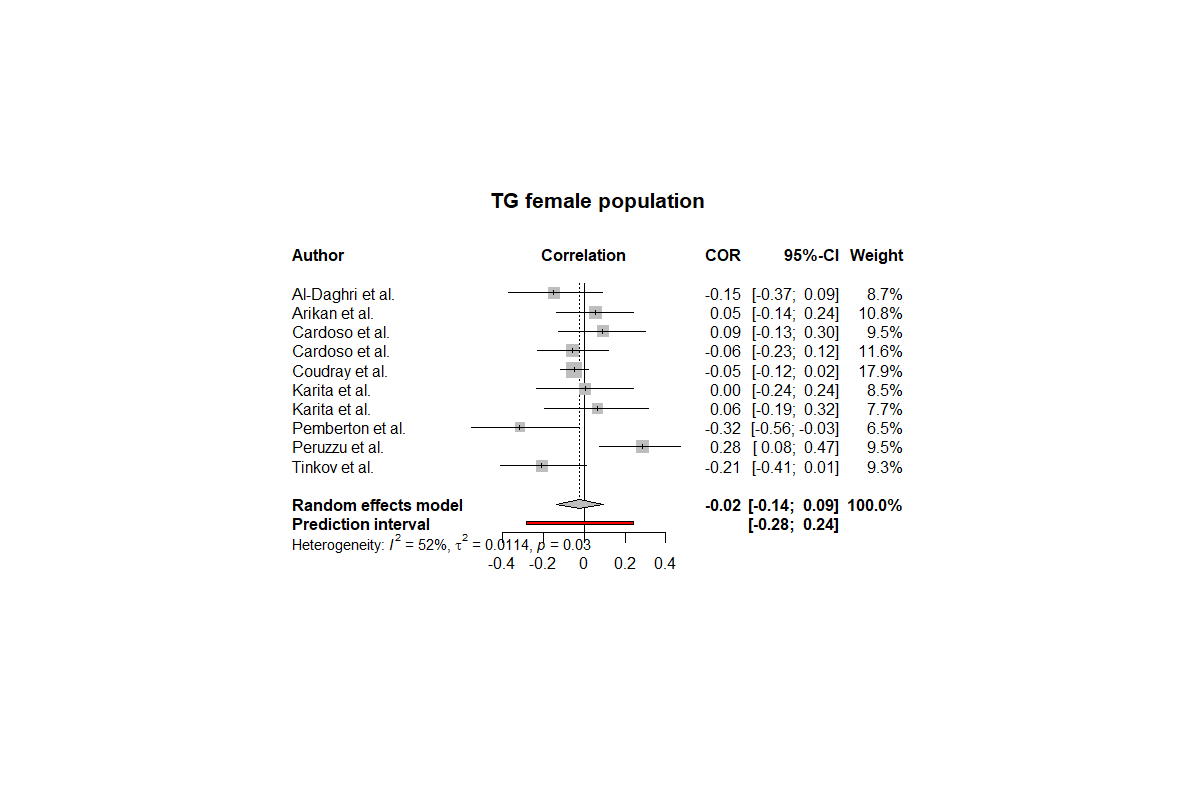


**Figure S5.** Pooled correlation between TG and serum selenium level in females.


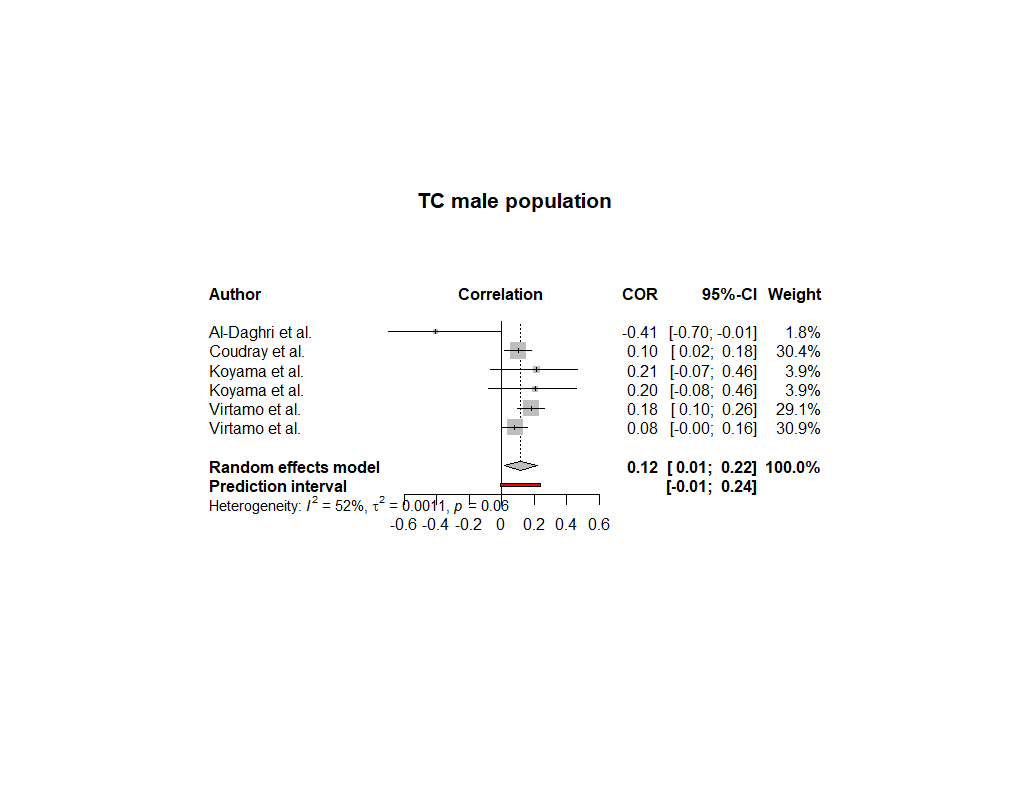


**Figure S6**. Pooled correlation between TC and serum selenium level in males.


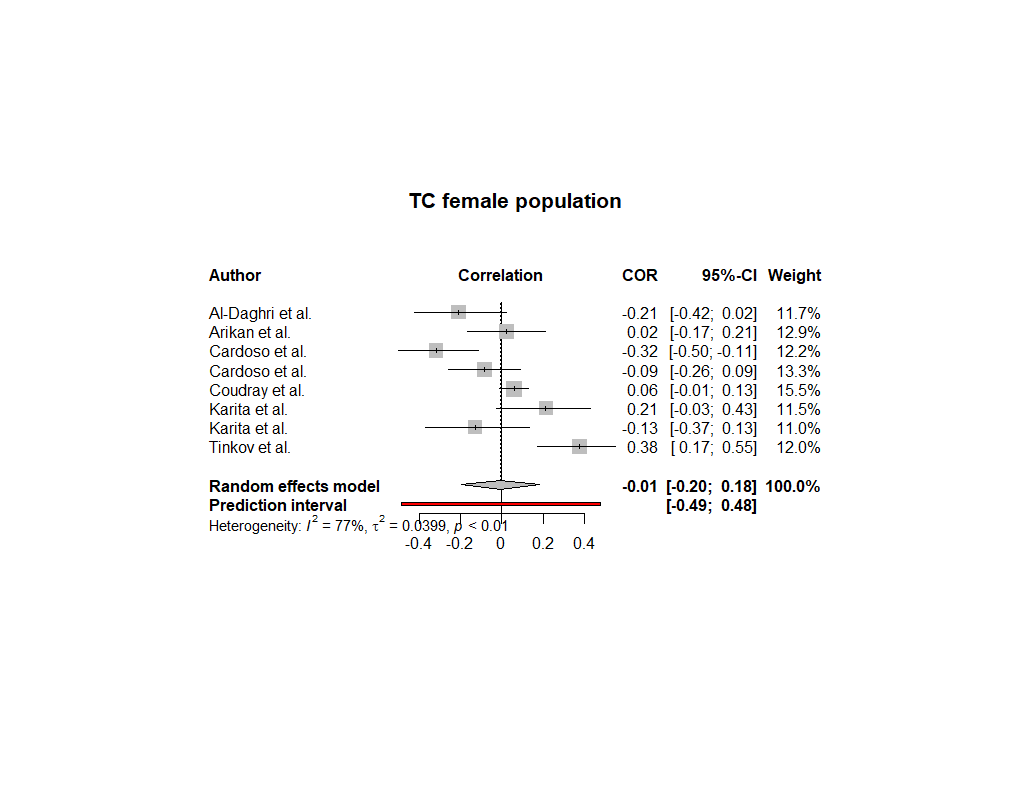


**Figure S7**. Pooled correlation between TC and serum selenium level in females.


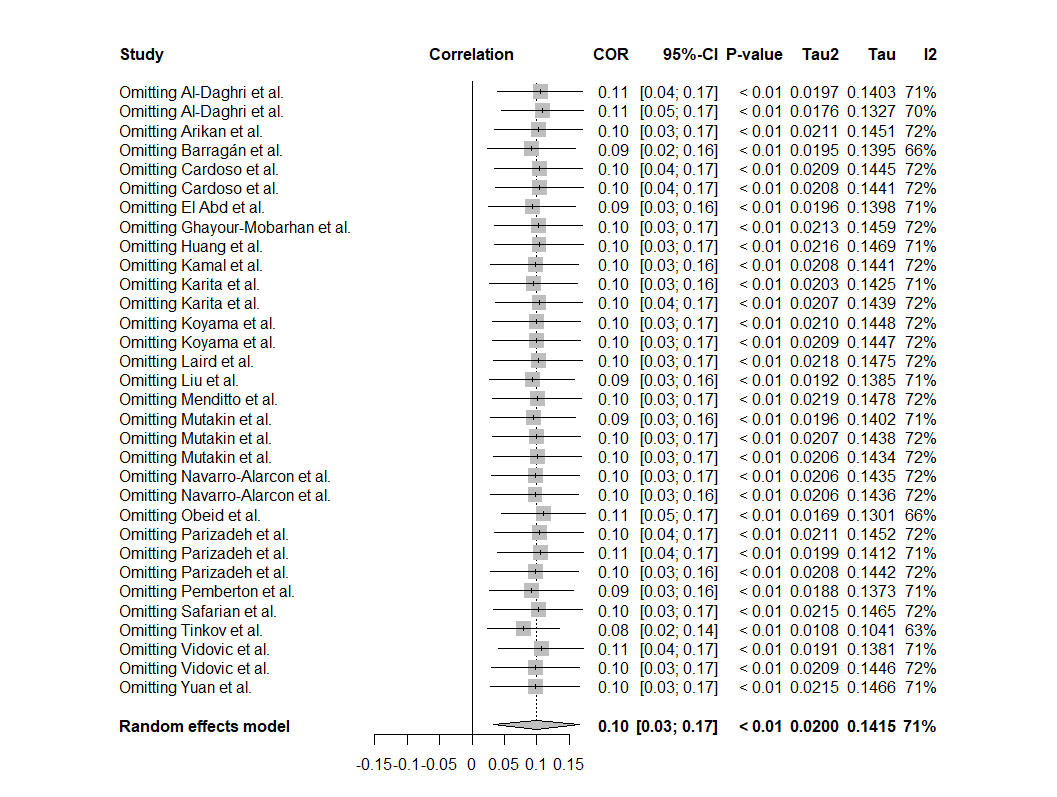


**Figure S8.** Leave-one-out sensitivity analysis correlation between HDL-C and serum selenium level among adults.


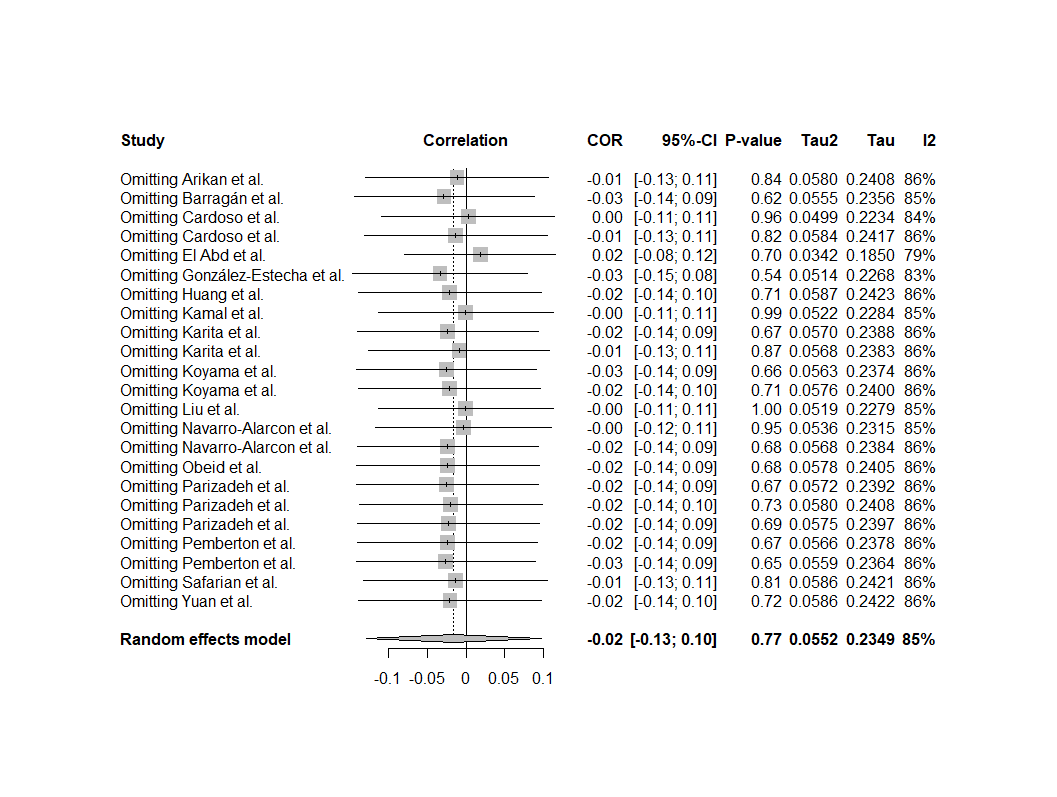


**Figure S9**. Leave-one-out sensitivity analysis correlation between LDL-C and serum selenium level among adults.


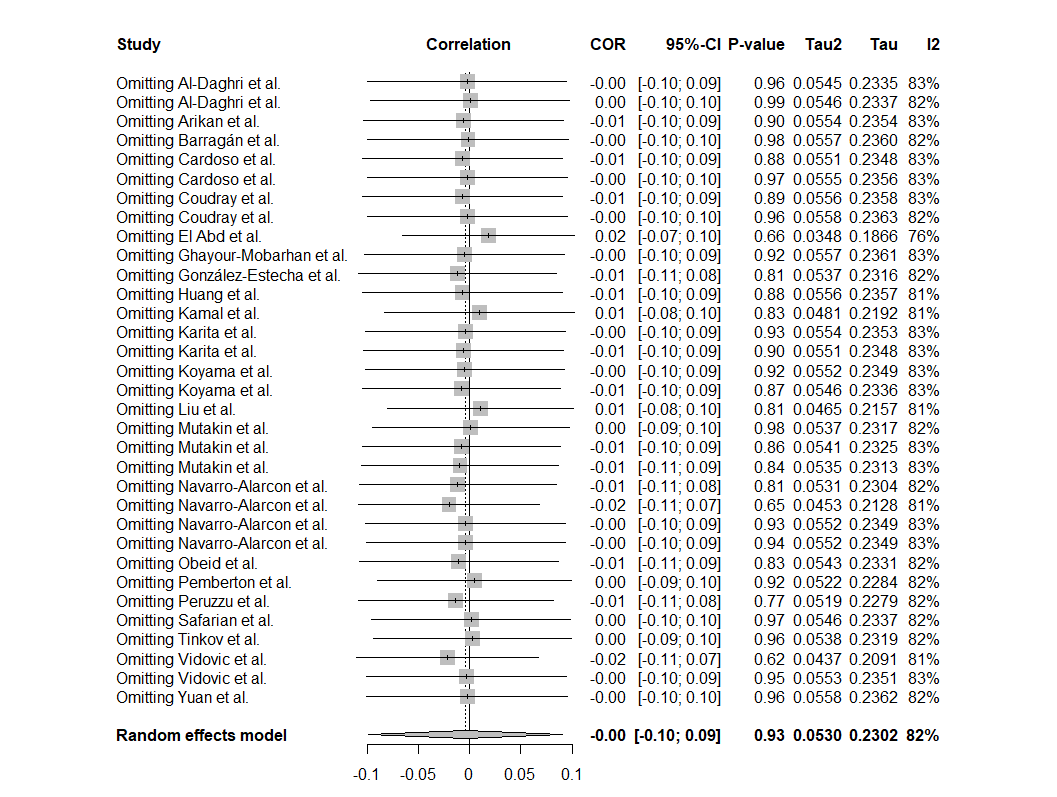


**Figure S10.** Leave-one-out sensitivity analysis correlation between TG and serum selenium level among adults.


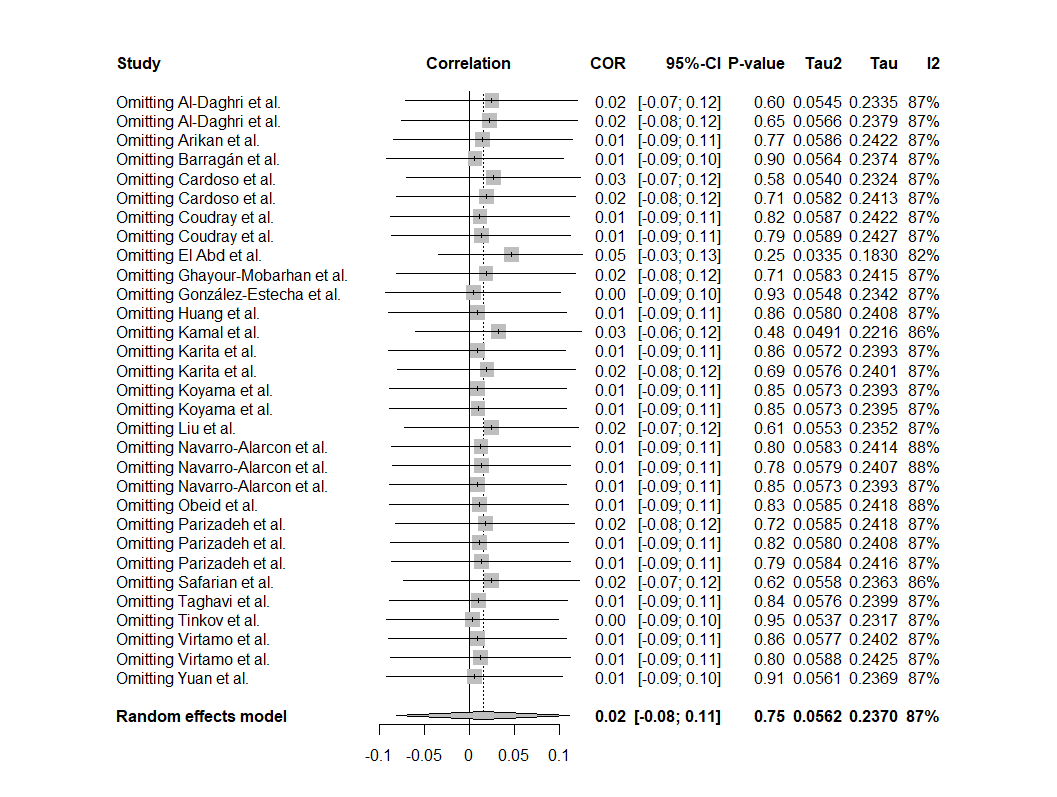


**Figure S11.** Leave-one-out sensitivity analysis correlation between TC and serum selenium level among adults.


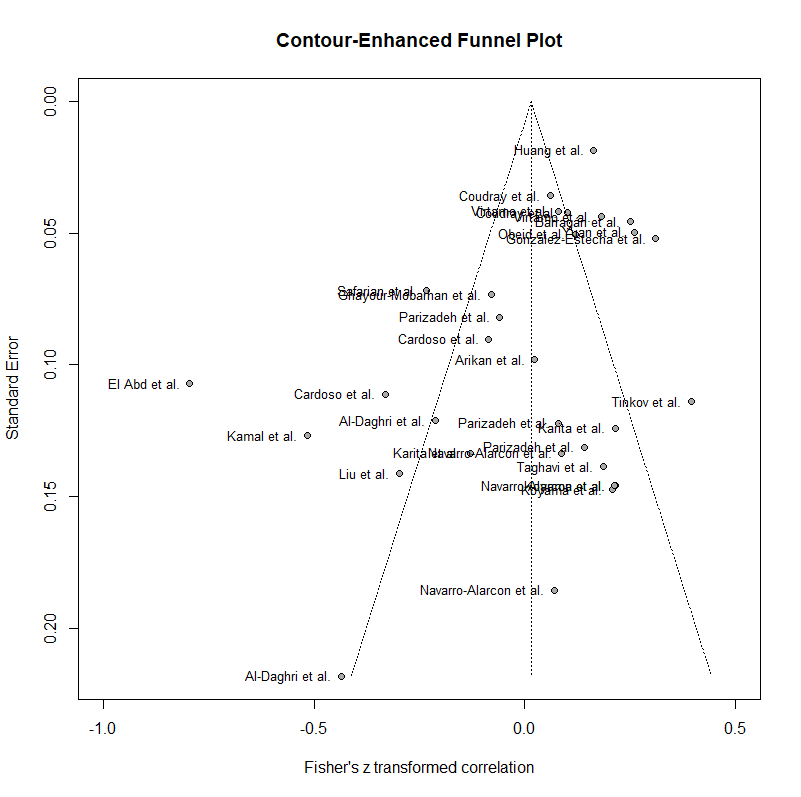


**Figure S12.** Funnel plot for publication bias of correlation between TC and serum selenium level among adults.


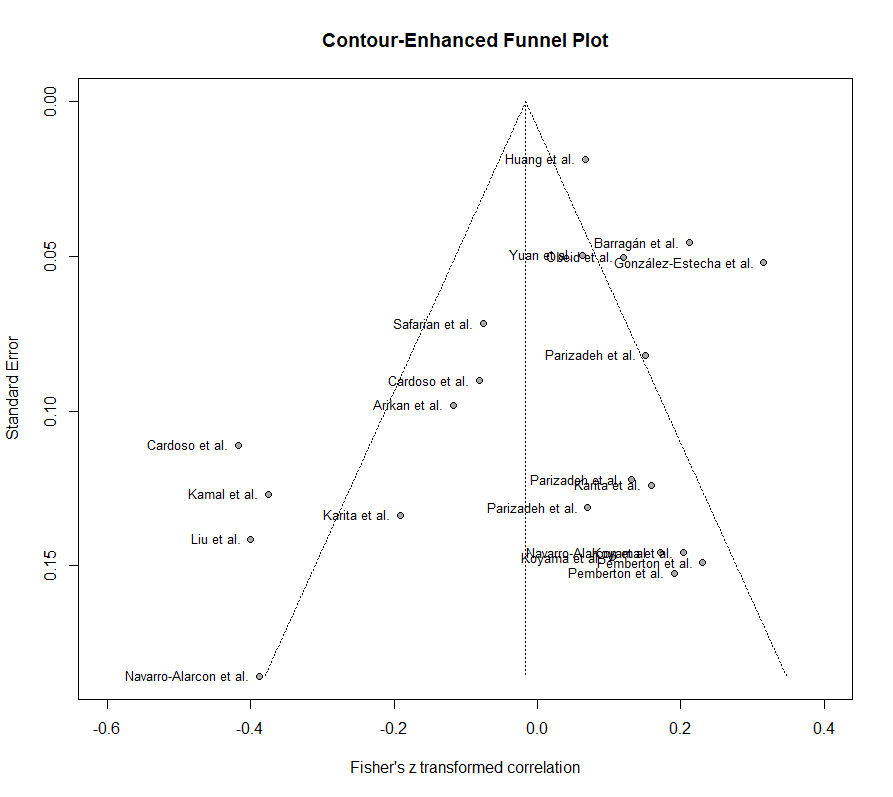


**Figure S13**. Funnel plot for publication bias of correlation between LDL-C and serum selenium level among adults.


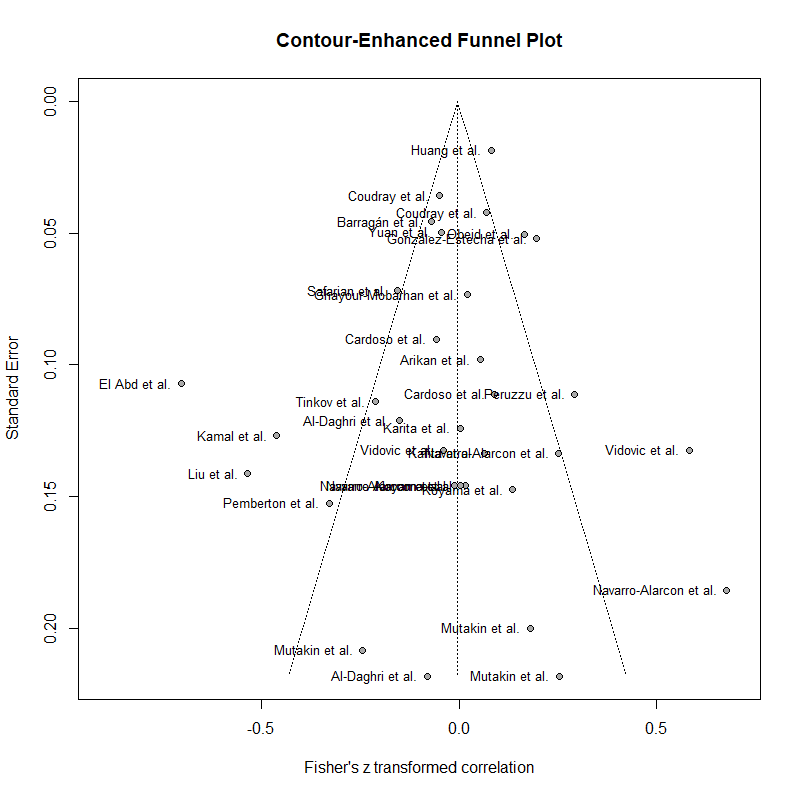


**Figure S14**. Funnel plot for publication bias of correlation between TG and serum selenium level among adults.


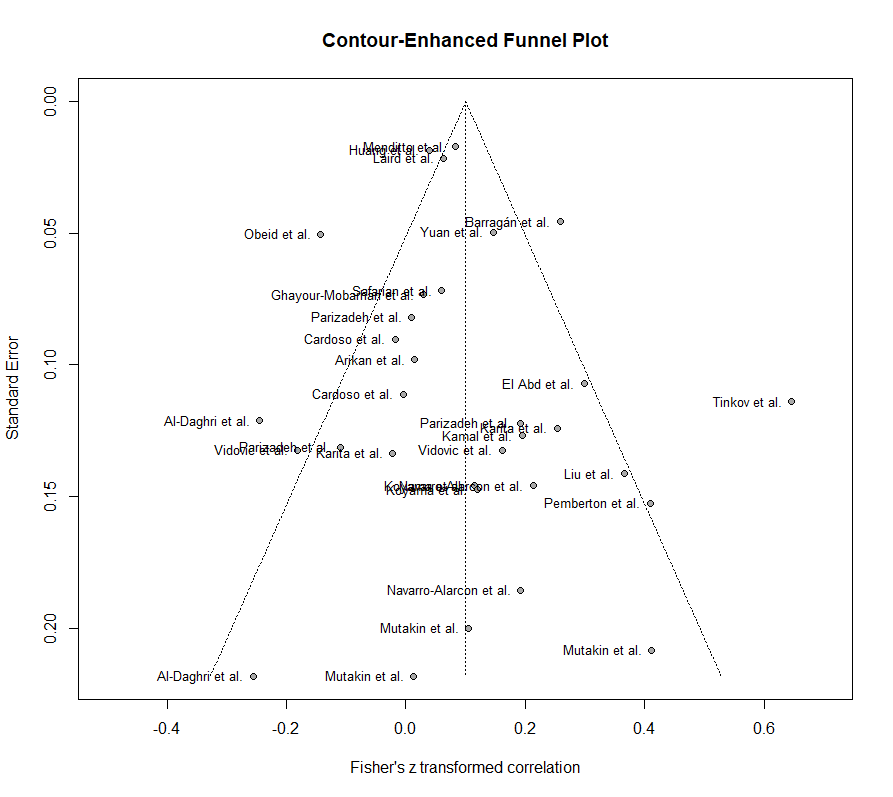


**Figure S15**. Funnel plot for publication bias of correlation between HDL-C and serum selenium level among adults.
